# Recent Advances in Lectin-Based Affinity Sorbents for Protein Glycosylation Studies

**DOI:** 10.3389/fmolb.2021.746822

**Published:** 2021-10-29

**Authors:** Anastasia Goumenou, Nathalie Delaunay, Valérie Pichon

**Affiliations:** ^1^ Department of Analytical, Bioanalytical Sciences and Miniaturization (LSABM), UMR 8231 Chemistry, Biology and Innovation (CBI), ESPCI Paris, CNRS, PSL University, Paris, France; ^2^ Sorbonne University, Paris, France

**Keywords:** lectins, selectivity, affinity, glycoproteins, glycopeptides, enrichment

## Abstract

Glycosylation is one of the most significant post-translational modifications occurring to proteins, since it affects some of their basic properties, such as their half-life or biological activity. The developments in analytical methodologies has greatly contributed to a more comprehensive understanding of the quantitative and qualitative characteristics of the glycosylation state of proteins. Despite those advances, the difficulty of a full characterization of glycosylation still remains, mainly due to the complexity of the glycoprotein and/or glycopeptide mixture especially when they are present in complex biological samples. For this reason, various techniques that allow a prior selective enrichment of exclusively glycosylated proteins or glycopeptides have been developed in the past and are coupled either on- or off- line with separation and detection methods**.** One of the most commonly implemented enrichment methods includes the use of lectin proteins immobilized on various solid supports. Lectins are a group of different, naturally occurring proteins that share a common characteristic, which concerns their affinity for specific sugar moieties of glycoproteins. This review presents the different formats and conditions for the use of lectins in affinity chromatography and in solid phase extraction, including their use in dispersive mode, along with the recent progress made on either commercial or home-made lectin-based affinity sorbents, which can lead to a fast and automated glycosylation analysis.

## Introduction

The increasing interest towards the characterisation of glycosylation is evident by the existence of numerous reports oriented towards the elucidation of the quantitative and qualitative characteristics of the glycoproteome (Xiao et al., 2019; Illiano et al., 2020). Indeed, glycosylation is considered as one of the most common and important post-translational modifications (PTM) of proteins and is associated with many essential intrinsic and extrinsic functions of proteins, including signalling, protein folding, interaction between proteins, cell migration and even alternation of the primary function of a given protein (Lis and Sharon, 1993; Schjoldager et al., 2020). Most importantly, the study of the glycoproteome can lead to the discovery of biomarkers related to a plethora of pathologies, since determination of protein glycosylation in biological fluids, tissues or cell culture extracts can serve as means of diagnosis and estimation of the progression of a disease (Pinho and Reis, 2015; Reily et al., 2019).

The use of lectins has been highly beneficial for the elucidation of the glycosylation state of a given sample. Lectins are proteins found in living organisms that can interact with specific sugar moieties of oligosaccharides attached to other biomolecules (Weis and Drickamer, 1996; Hirabayashi et al., 2015). Different analytical techniques that take advantage of the affinity of lectins towards oligosaccharides have been developed in the past. For example techniques like enzyme-linked lectin assay, lectin histochemistry, lectin blotting or lectin microarrays are based on the principles of other classical analytical methods, which are then adapted by the incorporation of lectin proteins and they can provide a quantitative and/or a qualitative glycan profiling of a sample (Dan et al., 2016; Hashim et al., 2017; Hendrickson and Zherdev, 2018).

Albeit the aforementioned techniques being extremely useful for the elucidation of the predominant glycosylation patterns in biological samples, other approaches such as mass spectrometry (MS) can provide an in-depth knowledge of the structural characteristics of glycoproteins (Morelle et al., 2006) and MS has thus become the most commonly used technique for the study of protein glycosylation. However, it is well established that especially in biological samples, the glycoproteins that can serve as disease biomarkers as an example, are usually of low abundance and are masked by other highly abundant proteins. Therefore, in order to “unmask” those glycoproteins of high importance but present at low concentration levels, an initial enrichment step is required. This process of enrichment can be performed at the glycoprotein level, or at the glycopeptide or glycan level after an enzymatic digestion, by implementing different techniques. For example, certain solid phase extraction (SPE) processes have been developed with solid supports functionalized with hydrazide or boronic acid derivatives, which are able to form covalent bonds with the glycan moieties (Chen C.-C. et al., 2014), or functionalized with anti-glycan antibodies specific to various glycan determinants (Cho et al., 2008). Additionally, chromatographic separations based on reversed phase (RP), size exclusion (SEC), ion exchange (IEC) or hydrophilic interaction liquid chromatography (HILIC), have been successfully used for glycosylation enrichment. All these techniques can be easily coupled with MS either off- or on-line. (Ongay et al., 2012; Huang et al., 2014; Riley et al., 2021).

Apart from the aforementioned techniques, enrichment can be performed with the aid of lectin-based affinity sorbents. This approach relies on the ability of the lectin protein to interact with specific glycosylation patterns present in glycoproteins. Immobilized lectins can be found in various formats, like on particles, magnetic beads or on monoliths to be used in capillary, cartridge or column setups and are applicable in SPE or in dispersive solid-phase extraction mode (dSPE). Another approach concerns the filter-assisted enrichment, where lectin-sorbents or free lectins are used in combination with a membrane of an appropriate molecular weight cut-off to separate the glycosylated forms from the non-glycosylated ones.

Lectin affinity enrichment can be advantageous compared to other techniques, as by choosing the appropriate lectin, only certain patterns of glycosylation can be enriched, i.e. only sialylation or fucosylation. This can lead to a reduction of the large heterogeneity of glycosylation for a more targeted enrichment. On the other hand, when a wider coverage of the glycoproteome is desirable, lectins with a broader selectivity can be used alone or in combination (Yang and Hancock, 2004; Madera et al., 2007; Kullolli et al., 2008). Lectin affinity extraction has been widely used for N-glycosylation enrichment and to a lesser extent for O-glycosylation studies (Durham and Regnier, 2006; Chalkley et al., 2009; Darula et al., 2012, 2016; Trinidad et al., 2012, 2013; Nagel et al., 2013; Vakhrushev et al., 2013; Darula and Medzihradszky, 2018). In this regard, a broader enrichment of both N- and O-glycosylation modifications can be achieved by using techniques like boronic acid chemistry or HILIC. Certain comparative studies have also shown that lectin affinity enrichment can lack in accuracy and sensitivity compared to other techniques (Zhang et al., 2016). However, it has been seen that the combination of the different techniques with lectin affinity enrichment can provide wider information for the glycosylation state of a sample (McDonald et al., 2009; Chen W. et al., 2014; Li et al., 2015; Zhou et al., 2017).

Overall, lectin affinity enrichment has been one of the most popular front-end approaches for the study of the glycosylation over the past decades. Given their very widespread use, the applicability of lectins in the study of the glycosylation has been extensively reviewed in the past (Monzo et al., 2007; Hirabayashi et al., 2011; Fanayan et al., 2012; Hage et al., 2012; Ongay et al., 2012; Alley et al., 2013; Huang et al., 2014; Ahn et al., 2015; Dan et al., 2016; Yamamoto et al., 2016; Hendrickson and Zherdev, 2018; Lastovickova et al., 2020; Wen et al., 2020). Those reviews are sometimes partially dedicated to lectin enrichment strategies and cover several aspects of the existing techniques. This review presents here an up-dated state-of-the-art of the use of commercial or home-made lectin-based affinity sorbents for extraction and enrichment of glycoproteins or glycopeptides. It presents also the progress that has been made in this field.

## Commercialized Lectin Affinity Sorbents

The high abundance of lectins in plant organisms, along with the existence of several techniques for their isolation and purification (Nascimento et al., 2012), renders them a relatively lower-cost solution for the development of lectin affinity sorbents, especially in comparison with sorbents functionalized with antibodies. For this reason, many companies, like Vector laboratories, EY laboratories, Sigma Aldrich, GE Healthcare etc., provide isolated purified lectins or lectins already immobilized on solid sorbents. In most of the cases, lectins in the commercialized affinity sorbents are immobilized on polymers, which are usually agarose based materials, such as Sepharose. Those materials are usually macroporous polymers, which is advantageous for lectin immobilization and trapping of large macromolecules. Additionally, they are generally stable over a wide range of pH (Zucca et al., 2016). Of course, the first criterion of choice of this kind of lectin-based sorbent is the nature of the lectin. Indeed, each lectin has its own specificity, as it is presented in Table 1, which includes the most commonly used lectins for the enrichment of glycoproteins and glycopeptides. Additionally, affinity-promoting metal ions as well as appropriate competitive saccharides or other conditions that may disrupt the lectin-glycan moiety bonds are depicted in this table. It should also be noted that one additional characteristic of lectins concerns the ability of certain classes of them to induce agglutination of blood cells and they can be therefore referred as agglutinins (Sharon and Lis, 2004).

**TABLE 1:** Most commonly used lectins for the enrichment of glycoproteins and glycopeptides.

Common Abbreviation	Source	Specificity	Competitive sugar or elution conditions/affinity-promoting metal ions	References
AAL	*Aleuria aurantia*	Fuc α1-6 [Core Fuc] > Fucα1-3, Fucα1-2	Fuc/−	Matsumura et al. (2007)
AOL	*Aspergillus oryzae*	Fuc α1-6 [CoreFuc] > Fuc α1-2 > Fuc α1-3, Fuc α1-4	Fuc/−	Matsumura et al. (2007)
Con A	*Canavalia ensiformis (concanavalin A)*	high-Man, hybrid type and complex biantennary N type	α-MM, α-MG/Mg^2+^, Mn^2+^, and Ca^2+^	Ogata et al. (1975)
DSL, DSA	*Datura stramonium*	β-GlcNAc: 2,6-branched complex tetra/triantennary > 2,4-branched complex tetra/triantennary N type	acidic conditions *, −	Kawashima et al. (1990)
ECL, ECA	*Erythrina Cristagalli*	Lactosamine: Gal β 1-4GlcNAc	Lac/Ca^2+^, Mn^2+^, and Zn^2+^	Wu et al. (2007)
GNL, GNA	*Galanthus Nivalis*	Core Man: monoantenary N type galacto&agalacto > bi/triantenary	α-MM/−	Shibuya et al. (1988)
HPA	*Helix pomatia*	T antigen	GlcNAc/−	Piller et al. (1990)
AIA	*Artocarpus integrifolia (Jacalin)*	Tn, T, sialyl T, and Core 3	Gal or Mel/−	Tachibana et al. (2006)
LcH,LCA	*Lens culinaris*	mono/biantenary N-type with Core Fuc [Fuc α1-6]	α-MM, α-MG/Ca^2+^, and Mn^2+^	Chandrasekaran et al. (2016)
LEL,TL	*Lycopersicon esculentum*	Polylactosamine: [(Gal β1-4GlcNAc)n, n ≥ 3], Lactosamine	acidic conditions/−	Kawashima et al. (1990)
LTL	*Lotus tetragonolobus*	Fuc α1-3/-2 (Gal β1-4)GlcNAc	Fuc/Ca^2+^, Mn^2+^	Bhattacharyya et al. (1990)
MAL I	*Maackia amurensis*	sialyllactosamine: Sia α2-3Gal β1-4GlcNAc	Lac/−	Knibbs et al. (1991)
MAL II, MAH	*Maackia amurensis*	disialyl-T	acidic conditions/−	Konami et al. (1994)
PHA-E	*Phaseolus vulgaris Erythroagglutinin*	Bisecting GlcNAc in galactosylated complex tetra/triantennary N-type	acidic conditions/Ca^2+^, Mn^2+^	Kaneda et al. (2002)
PHA-L	*Phaseolus vulgaris Leucoagglutinin*	Gal: 2,6-branched complex tetra/triantennary > complex bi antennary	acidic conditions, GlcNac/Ca^2+^, Mn^2+^	Kaneda et al. (2002)
PNA	*Arachis hypogaea (peanut aggluttinin)*	T, disialyl-T > core 2	Gal/Ca^2+^, Mg^2+^	Chandrasekaran et al. (2016)
PSA	*Pisum sativum*	mono/bianntenary N-type with Core Fuc [Fuc α1-6]	α-MM, α-MG/Ca^2+^, Mn^2+^	Tateno et al. (2009)
RCA I, RCA120	*Ricinus communis I*	Gal β1-4GlcNAc > Gal β1-3GlcNAc > core 2	Gal or Lac	Chandrasekaran et al. (2016)
SNA	*Sambucus nigra*	Sia α2-6Gal (NAc)-R	Lac, acidic conditions	Fischer and Brossmer, (1995)
UEA-I	*Ulex europaeus*	Fuc α1-2 [terminal] > Fuc α1-4 [subterminal]	Fuc/Ca^2+^, Mn^2+^, and Zn^2+^	Allen et al. (1977)
VVL, VVA	*Vicia villosa*	Tn	GalNAc/Ca^2+^, Mn^2+^	Puri et al. (1992)
WFA, WFL	*Wisteria floribunda*	GalNAc, GalNAc β1-4GlcNAc, Gal β1-3/-6Gal (NAc)	GalNAc, Lac/−	Piller et al. (1990)
WGA	*Triticum vulgaris (weat germ agglutinin)*	Chitin, GlcNAcβ 1-4GlcNAc, Sia (multivalent)	GlcNAc/Ca^2+^	Gallagher et al. (1985), Chandrasekaran et al. (2016)

Notes: α-MM: methyl α-D-mannopyranoside; α-MG: methyl α-D-glucopyranoside; Ca: calcium; Chitin: GlcNAc(β1−4)n; Core 2: GlcNAcβ1-6(Galβ1-3)GalNAc; Core 3: GlcNAc β1-3GalNAc-Ser/Thr; disialyl-T: [Sia α 2-3Galβ 1–3(Siaα2-6)GalNAc]-Ser/Thr; Fuc: fucose; Gal: galactose; GalNAc: N-acetylgalactosamine; Glc: glucose; GlcNAc: N-acetylglucosamine; Lac: lactose (Gal β1-4Glc) Man: mannose; Mel: melibiose; Mg: magnesium; Mn: manganese; Sia: Sialic acid; sialyl T: Sia α2-3Galβ1-3GalNAc- Ser/Thr; T: Galβ 1-3GalNAc-Serine(Ser)/Threonine(Thr); Tn: GalNAc-Ser/Thr; Zn: zinc.

In eukaryotes, proteins can be both N- or O-glycosylated (You et al., 2018; Schjoldager et al., 2020) and different lectins can have affinity towards the dominant motifs present in both of these types. For example, Concanavalin A (Con A), which is one of the most commonly used lectins, has affinity towards the trimanosyl core of N-glycans. However, Con A can only bind glycans with accessible mannose residues, meaning it can selectively capture oligomannose or hybrid type structures and to a lesser extend complex-type bi-antennary N-glycans, while it does not exhibit affinity for highly branched complex-type N-glycans (Ogata et al., 1975). Another commonly used lectin is the *Lens culinaris* lectin (LCA or LcH) (Chandrasekaran et al., 2016). This lectin is useful for the recognition of N-glycans that are core-fucosylated. *Aleuria aurantia* lectin (AAL) is also a lectin that is specific for core fucose (Matsumura et al., 2007). Wheat germ agglutinin (WGA) has been used for its ability to recognise N-acetyl glucosamine (N-GlcNac) oligomers and sialic acid (Sia) (Gallagher et al., 1985; Chandrasekaran et al., 2016). *Sambucus nigra* agglutinin (SNA) is also a sialic acid specific lectin (Fischer and Brossmer, 1995), while Thorn-apple agglutinin from *Datura stramonium* (DSA) is a lectin that has a higher affinity for tri- and tetra-antennary complex type N-glycans (Kawashima et al., 1990). The Jacalin lectin from *Artocarpus integrifolia* can be useful in the recognition of the Gal β1-3GalNAc epitope in O-glycans (Mucin type core 1) (Tachibana et al., 2006), while *Arachis hypogaea agglutinin* (PNA) also exhibits affinity towards the mucin type core 1, disialyl core 1 and the complex core 2 type (Chandrasekaran et al., 2016). Therefore, there are lectins that can be specific for both N- and O- glycosylation, and their affinity is dependent on the degree of antenarity, fucosylation, sialylation, and galactosylation etc of the glycans. Overall, the main advantages of commercially available lectin sorbents include that they are relatively low-cost and ready-to-use options, they offer a wide range of different glyco-epitope specificities and the used solid phases and are compatible with macromolecule extraction.

### Solid Phase Extraction With Commercial Lectin-Based Sorbents

As seen in Table 2, numerous authors have taken advantage of commercialized lectin sorbents for the study of the glycoproteome or for the purification of targeted glycoproteins. This table offers a summary of the studies conducted after 2012. Regarding preceding studies, the reader is invited to read the review of Ongay et al. (Ongay et al., 2012).

**TABLE 2:** Lectin solid phase extraction using commercial sorbents.

Lectin/provider	Format, volume, flow rate	Sample type, sample volume or amount	Processing before and during lectin extraction	Washing conditions	Elution conditions	Processing after lectin extraction	Objective	References
**Glycoproteins**								
AAL/Vector labs	cartridge, 600 μl	human serum, 300 μl	Depletion, isobaric labelling, dilution to 1 ml, incubation: 15 min	20 mM Tris pH 7.5, 5 V_C_	0.2 M Fuc, 4 x V_C_	tryptic digestion, LC-MS/MS	Intact protein-level labelling for quantitative analysis of serum glycoproteins	Nie et al. (2013)
AAL/Vector labs	spin column, 1 ml	human serum, 300 μl	Depletion, dilution to 1.7 ml, incubation: 16 h at 4°C	commercial buffer (N.S), N.S	commercial buffer (N.S), 50 μl	1) SDS-PAGE, in gel tryptic digestion 2) in-solution tryptic digestion	Quantitative, semi-quantitative and qualitative proteomic analysis: fucosylation	Ahn et al. (2014)
						LC-MS/MS		
AAL/Vector labs	cartridge, 500 μl	human plasma, 500 μl	Depletion, dilution (x2), incubation: overnight at 4°C	TBS (x5), N.S	0.1 M Fuc, 1 h incubation at 4°C, 10 x V_C_	SDS-PAGE,in-gel tryptic digestion, LC-MS/MS	Identification if fucosylation changes for biomarker discovery	Chang et al. (2019)
AAL PHA-L PHA-E/Vector labs	multi lectin cartridge, N.S	human serum, N.S	Depletion, dilution and isotopic labelling	N.S	Elution in series: AAL: 0.2 M Fuc PHA-L/E:0.1 M acetic acid pH 3.8	(RP) fractionation, tryptic digestion, LC-MS/MS	Quantitative proteomic analysis: core-fucosylation and highly-antenarity glycosylation	Totten et al. (2018)
Con A/GE Healthcare	cartridge, 2 ml	secretome of cell lines, 10 ml	incubation: overnight at 4°C	20 mM Tris pH 7.4, N.S	0.3 M α-MG	IEF, silver staining	Identification of core-fucosylated glycopeptides	Tan et al. (2014)
Con A GNA LcH/Qiagen	individual cartridges, 5 ml, 0.75 ml/min	extracted proteins from tomato fruit pericarp, 300 μg	N.A	20 mM Tris pH 7.0 + metal ions, 10 x V_C_	0.5 M α-MM, 5 x V_C_	tryptic digestion,RP-LC **1)** RP,LC-MS/MS **2)** HILIC, MS and MS/MS	Comparison of the coverage of the N-glycoproteome with different lectins	Ruiz-May et al. (2014)
Con A PNA WGA/Sigma-Aldrich	individual cartridges, 1 ml	lyophilized snake venom, 10 mg	incubation: 20 min	Con A/WGA: 20 mM Tris pH 7.4 + metal ions PNA: HEPES pH 8.0, 13 ml	Con A:0.5 M Gluc WGA: buffer pH 3.0 with 0.5 M GlcNAc PNA: buffer pH 3.0 with 0.5 M GlcNAc, 5 ml	protein precipitation, tryptic digestion, LC-MS/MS	Bottom-up analysis of total proteome and glycoproteome	Andrade-Silva et al. (2016)
Con A/GE healthcare DSA/J-Oil Mills WFA/J-Oil Mills	Individual cartridges, 1 ml	cell culture medium &seminal plasma	incubation: 20 minat 4°C	Con A: TBS pH 7.4 DSA/WFA: PBS pH 7.4 + metal ions,4.75 ml	Con A: 0.3 M α-MG DSA: acidic solution (0.1 M acetic acid +0.1 mg/ml BSA) WFA: 0.4 M Lac, 5 ml	thermolysin digestion, MALDI-MS^n^	Glycan profiling for biomarker identification	Ideo et al. (2020)
LEL/Vector labs HPA/EY laboratories	Individual columns in tandem, 50 × 4.6 mm, 0.3 ml/min	human plasma, 100 μg	immunosorbent enrichment (in series)	0.10 M HEPES pH 7.5 + metal ions	0.5 M acetic acid pH 2.5	tryptic digestion, PNGase F, LC-MS/MS	enrichment of glycosylated proteins	Jung and Cho, (2013)
PHA-E	cartridge, N.S	depleted human serum	incubation: overnight at 4°C	10 mM Tris + metal ions	0.2 M GlcNAc	SDS-PAGE and in-gel tryptic digestion, LC-MS/MS	Biomarker identification after lectin enrichment	Liu et al. (2016a)
VVA/Vector labs	cartridge, 8 ml	fallow deer placental tissue, 80 mg	ammonium sulphate precipitation, incubation: overnight at RT	0.001 M HEPES pH 7.6, 80 ml	0.05 M GalNAc	SDS-PAGE, western blot and N-terminal sequencing	Isolation and characterization of pregnancy hormones	Bériot et al. (2014)
VVA/Vector labs	cartridge, 300 μl	whole proteins from cell lines	Neuraminidase	1) 0.4 M Gluc in 20 mM Tris pH 7.4 + metal ions, 10 x V_C_ 2) 50 mM ammonium bicarbonate, 1 ml	RapiGest (90°C for 10 min), 500 μl (x4)	tryptic digestion, LC-MS/MS	localization of STn overexpression in cancer glycoproteins	Peixoto et al. (2016)
VVA - agarose/Vector labs	cartridge, 300 μl	human bladder tissue sections, 1 mg of total protein	PNGase F, neuraminidase	1) 0.4 M Gluc in 20 mM Tris pH 7.4 + metal ions, 10 x V_C_ 2) 50 mM ammonium bicarbonate, 1 ml	RapiGest (90°C for 10 min), 500 μl (x4)	tryptic digestion, LC-MS/MS	O-glycosylation proteomic analysis in cancer	Cotton et al. (2017)
Con A,WGA Jacalin/GE Healthcare	multiple lectin cartridge, mix of 0.5 ml of each lectin-sorbent	depleted human plasma, 100 μl	dilution to 1 ml, incubation: 2 h at 4°C	20 mM Tris pH 7.4 + metal ions (x3)	0.2 M α-MM, 0.2 M α-MG, 0.5 M GlcNAc and 0.8 M Gal, 5 ml	SDS-PAGE, IEF, in gel-tryptic digestion, MS and MS/MS	Glycoproteome analysis for biomarker discovery	Bag et al. (2014)
**Glycopeptides**								
AAL/J-Oil Mills	column, 50 × 5 mm	proteins from kidney homogenates of mice	tryptic digestion, HILIC	10 mM Tris pH 7.5	0.005 M Fuc (4x)	1) PNGase, IGOT-LC-MS 2) PNGase, permethylation, MALDI-MS 3) Desialylation, LC-MS	Recognition of Lewis x motif in the glycoproteome	Noro et al. (2015)
Con A WGA RCA I/Sigma Aldrich	Individual cartridges alone,33 μl of Con A, 28.55 μl of WGA and 50 μl of RCA I	Mouse brain tissue, 200 μg (tryptic digest)	1and2) tryptic digestion 3) tryptic digestion, HILIC enrichment incubation: overnight at 4°C	N.A	50 mM ammonium bicarbonate in 10% (v/v) ACN and 50 mM ammonium bicarbonate, 100 μl (6x) (centrifugation)	1) PNGase F in H_2_^18^O 2) HILIC enrichment/PNGase Fin H_2_^18^O 3) PNGase F in H_2_^18^O LC-MS/MS	Comparison of enrichment methods for N-Glycosylation site localization	Zhang et al. (2016)
LCA/Vector labs	Spin column, 1 ml	human sera, 40 μl (tryptic digest)	Depletion, tryptic digestion, dilution (460 µl), labelling, incubation: 20 min at RT	20 mM Tris pH 7.4, 4 ml	0.2 M α-MG 0.2 M α-MM, 800 μl tight bound peptides: saccharide solution without NaCl or metal ions/3 × 800 μl	partial deglycosylation (Endo F3), LC-MS/MS	Localization of core-fucosylated glycopeptide sites	Tan et al. (2015)
PNA VVA/Vector labs	Individual columns,PNA: 800 μl, VVA: 300 μl,column, 2.6 m × 1.5 mm, 100 μl/min	total cell lysate or secretome of cell lines	1) VVA column 2) neuramidase treatment, PNA column	N.S	short VVA:RapiGest 4x (90°C for 10 min) VVA/PNA LWAC and short PNA	1) tryptic or chymotrypsin and Glu-C, VVA LWAC 2) tryptic digestion, PNA LWAC, VAA LWAC isoelectric focusing, nano-LC MS	GalNAc-type O-Glycoproteome analysis	Yang et al. (2014)
PNA VVA/Vector labs	column 2.6 m × 1.5 mm, 100 μl/min	human platelets,endothelial cells, plasma	Neuramidase, trypsin or chymotrypsin digestion	PNA: 10 mM HEPES pH 7.4 VVA: 20 mM Tris pH 7.4 + metal ions	PNA: 0.5 M Gal, 1 x V_C_+ 1 M Gal,2 x V_C_ VVA: 0.2 M GalNAc, 2 x V_C_ + 0.4 M GalNAc, 1 x V_C_	LC-MS/MS	Localization of O-glycans in plasma and blood cells	King et al. (2017)
Con A LCH WGA/N.S	N.S	Model glycoproteins and serum	serum depletion, tryptic digestion	N.S	N.S	labelling, LC-MS/MS	Automated Quantitative analysis of enriched glycopeptides after labelling	Wang et al. (2014)
SNA MAL II—AffiSep^®^/Galab Technologies	spin column, 150 μl	Depleted human serum, 40 μl (tryptic digest)	tryptic digestion, 1:10 dilution, incubation: overnight at 4°C	SNA: 10 mM HEPES pH 8.0, 600 μl (x2) MAL II: 10 mM HEPES pH 7.5	SNA:0.2 M Lac, 200 μl + 0.2 M Lac in 0.2 M acetic acid, 200 μl MAL II:0.2 M Lac, 2 × 200 μl	LC-MS/(MS)	Qualitative and quantitative changes in sialylation for biomarker discovery	Kontro et al. (2014)

NOTES: Endo F3: endoglycosidase F3; FA; formic acid; HILIC; hydrophilic interaction chromatography; IEF: isoelectric focusing; LC: liquid chromatography; LWAC: lectin weak affinity chromatography; MALDI; matrix-assisted laser desorption/ionization; MS; mass spectrometry; N.A: not applicable; N.S: not specified; PNGase F: peptide N-glycosidase F; RP: Reversed phase; RPLC: Reverse phase liquid chromatography; RT: room temperature; SDS-PAGE: sodium dodecyl sulphate–polyacrylamide gel electrophoresis; STn: cell-surface Sialyl-Tn antigen; TBS: tris-buffered saline.

**FIGURE 1:**
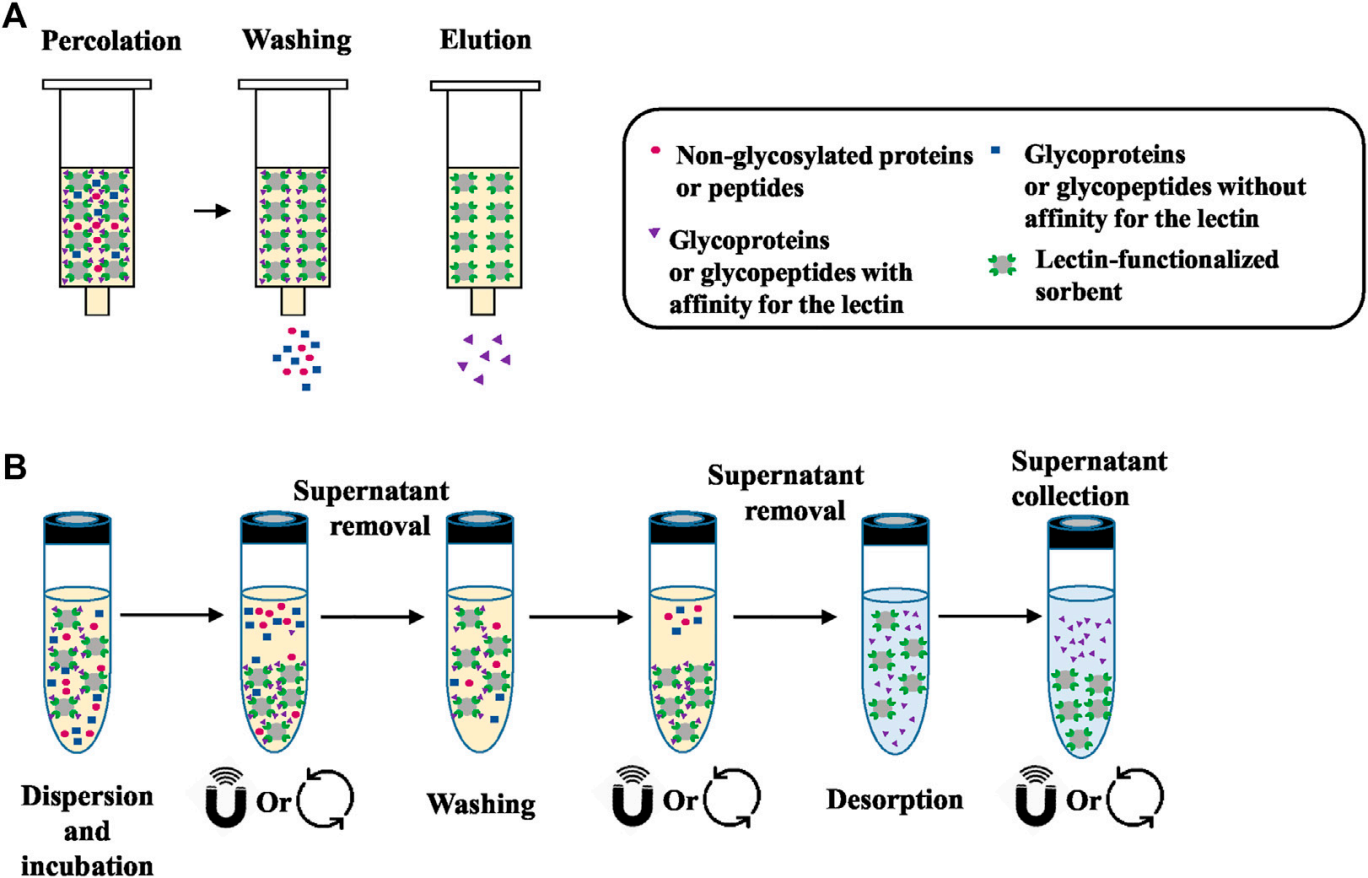
**(A)** General workflow of lectin SPE. Initially the sample, which is a mixture of molecules with and without affinity for the lectin sorbent, is percolated through the cartridge. Percolation is followed by washing of the components that do not have affinity and finally elution of the target glycoproteins or glycopeptides by disrupting the lectin-sugar bonds. **(B)** General workflow of lectin dSPE. Initially the sample is mixed “in solution” with the lectin-functionalized sorbent in order to capture those molecules that have affinity with the lectin. After centrifugation or the application of a magnetic field, the compounds that do not have affinity are collected from the supernatant. Additional washing steps can be implemented to remove all non-retained compounds. The final desorption of target glycoproteins or glycopeptides is performed under the appropriate conditions (see Table 3).

The technique used in these studies is mainly referred as lectin affinity chromatography (Kobata and Endo, 1992) or lectin affinity enrichment where the lectin-based sorbent is packed in a cartridge or a column and it adopts the principles of a SPE process. A general workflow of lectin SPE is depicted in Figure 1A **.** The usual experimental protocol of lectin SPE includes an initial conditioning step of the lectin sorbent followed by percolation of the sample, washing of the non-reactive components and finally elution of the target glycoproteins or glycopeptides by disrupting the lectin-carbohydrate bonds. The percolation, washing and sample dilution buffers have in the majority of the cases the same composition and are in general physiological pH solutions with medium ionic strength. Additionally, as seen in Tables 1, 2, some lectins, like Con A, necessitate the presence of metal ions like manganese, magnesium or calcium (Mg^2+^, Mn^2+^ or Ca^2+^) in concentrations of 0.5–1 mM to exhibit their affinity (Brewer et al., 1983). The dissociation of the trapped forms seems to be achieved always rapidly by applying a buffer that usually has again the same composition as the washing buffer but with the addition of the competitive sugar having a strong affinity towards the lectin. However, in some cases acidic solutions with or without the presence of the competitive sugar were implemented. Interestingly, in one study it was seen that the efficiency of the elution of the core fucosylated LCA-captured glycopeptides from sera was increased when the metal ions and NaCl, which contributes to the ionic strength of the buffer, were eliminated from the saccharide solution for elution (Tan et al., 2015).

Almost in the totality of the studies mentioned in Table 2, lectin-based SPE was combined with an MS glycoproteomic analysis of biological samples of various origins. In this table it is also seen that 2/3 of those studies concerned an enrichment process performed at the glycoprotein level, meaning that the intact proteins were subjected to lectin SPE. However, the overall analytical workflow usually includes further sample handling processes like labelling, HILIC, gel electrophoresis etc. followed or preceded by a step of digestion and/or de-glycosylation before (LC)-MS identification and/or quantification. On the other hand, enrichment can be performed at the glycopeptide level, where an initial digestion of the sample is performed before application to the lectin sorbent.

Lectin-based SPE can be performed by gravity flow (Nie et al., 2013; Bag et al., 2014; Bériot et al., 2014; Tan et al., 2014; Yang et al., 2014; Noro et al., 2015, 2015; Liu et al., 2016a, Liu et al., 2016b; Andrade-Silva et al., 2016; Peixoto et al., 2016; Zhang et al., 2016; Cotton et al., 2017; Azevedo et al., 2018; Chang et al., 2019; Ideo et al., 2020) or with low-pressure conditions (Jung and Cho, 2013; Yang et al., 2014, 2014; Ruiz-May et al., 2014; King et al., 2017; Totten et al., 2018). However, spin column formats have also been proposed, which necessitate a centrifugation step in order to perform the washing and elution steps (Ahn et al., 2014; Kontro et al., 2014; Tan et al., 2014, 2015; Zhang et al., 2015). The size of the cartridge/column is usually dependent on the amount of sample applied. However, the exact protein content of the applied sample is generally not specified. For glycoprotein enrichment, cartridges containing the lectin-based sorbent with volumes from 0.3 ml (Peixoto et al., 2016; Cotton et al., 2017) to 8 ml (Bériot et al., 2014) were used, while at the glycopeptide level, volumes as low as 28 μl (Zhang et al., 2016) or very large columns as the one of 2.6 m length and 1.5 mm internal diameter (i.d.) (Yang et al., 2014) were reported.

It is worthwhile noticing that an incubation time, i.e. a contact time between the sample and the lectin-based sorbent, is often introduced after the percolation of the sample, whether glycoproteins or glycopeptides were aimed to be trapped. This incubation can last a few minutes (Nie et al., 2013; Tan et al., 2015; Andrade-Silva et al., 2016; Ideo et al., 2020), some hours (Ahn et al., 2014; Bag et al., 2014) and in one third of cases overnight (Bériot et al., 2014; Kontro et al., 2014; Tan et al., 2014; Liu et al., 2016a; Zhang et al., 2016; Chang et al., 2019), which is quite unusual in a conventional SPE process. Indeed, this step greatly increases the analysis time, but the necessity of this interaction time for the enhancement of glycan moieties absorption was only clearly stated in one study (Kontro et al., 2014). Some groups do not report any interaction time at all (Jung and Cho, 2013; Ruiz-May et al., 2014; Yang et al., 2014; Noro et al., 2015; Peixoto et al., 2016; Cotton et al., 2017; King et al., 2017; Totten et al., 2018).

In regard to the elution step, the elution solution volume used is dependent on the size of the cartridge. Most research groups applied about 5–10 column volumes of the appropriate buffer, which is usually deemed sufficient for elution. It can be noticed that the elution volume is often significantly superior to the initial sample volume. This reflects a purification of targeted glycoproteins or glycopeptides rather than a concentration step and as a result, most research groups proceed with further concentration and/or desalting of the eluents with the use of specific filters. This buffer exchange process is also often necessary due to the incompatibility of the buffers used to disrupt the interactions between the lectins and the glycan moieties with MS, since they can contain salts, metal ions and sugars. As an example, glycoproteins, originally contained in 300 μl of depleted and isobaric labelled serum diluted with 1 ml of binding buffer, were extracted on a 600 μl volume fucose-specific AAL cartridge. The elution was performed with 4 cartridge volumes (Vc) of the saccharide buffer, leading to a larger final volume after extraction (Nie et al., 2013). Similarly, 500 μl of depleted serum that were further diluted two times were percolated on a 500 μl AAL-cartridge and retained proteins were further eluted with 10 Vc of buffer (Chang et al., 2019). In both cases, the eluents were further processed with concentration and buffer exchange filters. Interestingly, a desorption of the glycoproteins with only 2 × 50 μl of the saccharide buffer was reported to recover them from a 300 µl depleted serum sample diluted to 1.7 ml before passing through the lectin sorbent (Ahn et al., 2014).

Apart from the use of single lectin cartridges, the mixture of lectin-affinity sorbents offers the opportunity of a multi-lectin extraction process (Yang and Hancock, 2004). For example, 0.5 ml of each Con A-, WGA-, and Jacalin-agarose sorbents were mixed in a single column, and after the percolation of 100 μl of depleted plasma diluted to a volume of 1 ml, the retained glycoproteins were eluted with 5 ml of a buffer containing the 3 corresponding competitive sugars for each lectin (Bag et al., 2014). Since those lectins have a broad specificity (see Table 1), a greater coverage of the glycoproteome was achieved.

**FIGURE 2:**
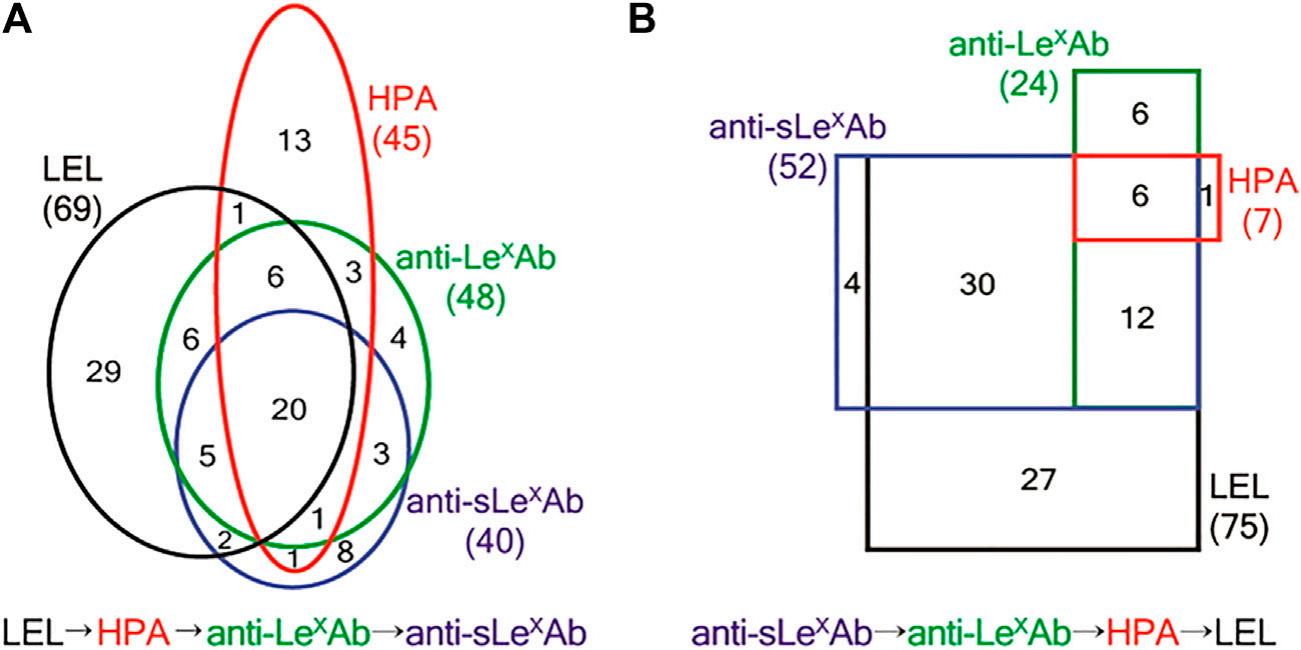
**(A)** Number of proteins identified from LEL → HPA → anti-LexAb → anti-sLexAb SAC. Numbers in the black, red, green, and blue ovals show the proteins identified from LEL, HPA, anti-LexAb, and anti-sLexAb columns, respectively. A total of 102 proteins were identified. **(B)** Numbers of proteins identified from anti-sLexAb → anti-LexAb → HPA → LEL SAC. Numbers in the black, red, green, and blue rectangles show the proteins identified from LEL, HPA, anti-LexAb, and anti-sLexAb columns, respectively. A total of 86 proteins were identified. LEL: *Lycopersicon esculentum* lectin. HPA: *Helix pomatia* lectin. anti-LexAb: Anti-Lewis x antibody. anti-sLexAb: anti sialyl Lewis x antibody. SAC: serial affinity chromatography. Reprinted with permission from Jung, K., and Cho, Anal. Chem. 2013, 85, 7,125–7,132, Copyright ^©^ 2013, American Chemical Society.

Finally, it should be noted that the focus of the studies mentioned in Table 2 is usually the proteomic analysis after lectin extraction. Therefore, the characteristics of the lectin cartridges, like the capacity of the lectin sorbent or the recovery after enrichment are most of the times not mentioned. It was therefore usually not possible to evaluate the performance characteristics of the lectin cartridges independently from the entire analytical procedure or retrieve information regarding the recovery after lectin SPE. Nevertheless, lectin enrichment offers a valuable initial sample “clean-up” step, to retrieve specifically only glycosylated forms out of the large pool of other interfering non-glycosylated proteins and/or of those glycosylated forms that do not have affinity for the selected lectin. This extraction step can potentially reduce the matrix effects of the sample due to the presence of interfering forms and therefore enhance the detectability of low abundance targeted glycoforms and allow the detection of a large number of glycoproteins and/or glycosylation sites in biological samples (Jung and Cho, 2013; Nie et al., 2013; Ruiz-May et al., 2014; Yang et al., 2014; Noro et al., 2015; Tan et al., 2015; Andrade-Silva et al., 2016; Bu et al., 2017; King et al., 2017). As an example, the thorough mining of the O-glycoproteοme of the hemostatic system was achieved by using a technique called lectin weak affinity chromatography (LWAC) combined with higher-energy collisional dissociation (HCD) and electron transfer dissociation (ETD) LC-MS/MS (King et al., 2017). In LWAC, the lectin-sorbent material is packed in a relatively large column (2.6 m × 1.5 mm), while enrichment is performed at a low flow rate of 100 μl/min. This extended process is known to enhance the lectin-sugar moiety interactions and can increase the specificity of enrichment (Ma et al., 2013). Therefore, in the study of King et al., a LWAC format was implemented with the use of PNA and *Vicia Villosa* (VVA) lectins, which are known to have affinity towards O-glycosylation. Two lectin columns, each one packed with agarose functionalized either with PNA or VVA sorbent, were used in sequence and as a result, the largest dataset, as compared to previous studies, of O-glycoproteins and O-glycosites originating from endothelial cells, platelets and plasma was established, thus highlighting the great potential of lectin enrichment for glycoproteome elucidation. In another interesting study, a protocol combining lectin columns coupled in series with antibody columns in a low pressure system setup was implemented (Jung and Cho, 2013). Two lectin columns of 0.8 ml, one functionalized with a *Lycopersicon esculentum* lectin (LEL) and the other with a *Helix pomatia* lectin (HPA), were placed in-series either in front or after two anti-Lewis x [Gal β1-4(Fuc α1-3)GlcNAc] and anti-sialyl Lewis x [Siaα2-3Galβ1-4(Fucα1-3)GlcNAc] immunoglobulin M (IgM) affinity sorbents. The complementarity of the two different setups was shown, since distinct glycosylation capturing coverages were observed when the lectin columns were placed in front of the antibodies (Figure 2A) and vice versa (Figure 2B). Additionally, multiple studies were conducted for the identification and/or monitoring of the up and down-regulation of the glycoproteins that can be used as biomarkers for disease progression and diagnosis with lectin enrichment combined with MS quantitative studies (Ahn et al., 2014; Kontro et al., 2014; Tan et al., 2014; Liu et al., 2017; Totten et al., 2018; Chang et al., 2019; Ideo et al., 2020). These observations are indicative of the utility of lectin enrichment in the improvement of the qualitative and/or quantitative data obtained. However, caution should always be taken when evaluating the data after lectin SPE, since false identifications of glycosylation sites have been noted (Zhang et al., 2016).

### Dispersive Solid Phase Extraction With Commercial Lectin-Based Sorbents

A summary of the studies where enrichment is performed “in solution” using commercially available lectin functionalized sorbents is presented in Table 3. A general workflow of the dSPE lectin enrichment is depicted in Figure 1B, where it is seen that instead of percolating the sample though the lectin column, it is mixed in direct contact with the lectin-sorbent. This allows the capturing of the compounds that exhibit affinity with the lectin. As seen in Table 3, an overnight incubation step between the sample and the lectin sorbent is usually performed. The unbound fraction is then collected from the supernatant usually after centrifugation. In the case where magnetic particles functionalized with lectins are used, a magnetic field is applied. Additional washing steps are usually incorporated to ensure adequate removal of the non-retained components of the sample, while the final desorption of the targeted compounds is performed with the appropriate buffer and/or with heating. It is worthwhile noticing that the nature of the binding, washing and elution solutions described in Table 3 is very close to the ones used in the corresponding steps in the SPE process (Table 2). Indeed, they are the same ones that promote or disrupt the interactions between the lectins and their corresponding targets. While the recovery of glycosylated proteins or peptides may appear tedious due to the need for centrifugation steps in dSPE, the fact that no membranes or frits are used, as in the case of cartridges or columns, eliminates the risk of clogging during the percolation of biological samples.

**TABLE 3:** Lectin dispersive solid phase extraction using commercial sorbents.

Lectin/provider	Affinity sorbent amount	Sample, sample volume or amount	Processing before and during the extraction on lectin	Washing conditions	Desorption conditions	Processing after lectin desorption	Objective	References
**Glycoproteins**								
AAL, SNA/Vector labs	60 μl SNA and 90 μl AAL	human serum	Depletion, incubation: overnight at 4°C	50 mM PBS pH 7.5, 200 µl (x5)	100 mM acetic acid, 400 µl	tryptic digestion, LC-MS/MS	Comparison of lectin enrichment with hydrazide chemistry for biomarker discovery	Song et al. (2014)
AAL, WGA, SNA/Vector labs	intact proteins: 250 μl lectin sorbent glycopeptides: 100 μl lectin sorbent	human serum, 25 μl	dilution at 1:10 in buffer, incubation: overnight at 4°C	200 mM Tris buffer pH 7.5 (x3)	AAL: 0.1 M Fuc SNA: 0.5 mM Lac WGA: 0.8 M N-GlcNAc in 0.2 M acetic acid, 100 μl, 30 min	tryptic digestion, second lectin enrichment, hydrazide enrichment, PNGase F LC–MS/MS	comparison of three approaches for enrichment of the glycoproteome of human sera	Li et al. (2015)
Con A, WGA, Jacalin/Vectorlabs	mix of 200 μl	human follicular fluid	incubation: overnight at 4°C	PBS (1x)	Con A: 0.2 M α-MM WGA: 0.5 M GlcNAc JAC: 0.1 M Mel, 30 min incubation, 10 min centrifugation at 4°C (x2)	tryptic digestion, labelling, SCX, SAX stage tip fractionation, LC-MS/MS, immunoblotting	glycoproteome study for biomarker discovery	Patil et al. (2019)
VVA	N.S	organelles from Toxoplasma gondii	parasite lysate in RIPA buffer	50 mM Tris pH 7.4 + metal ions (x5)	GalNAc	SDS-PAGE, in-gel tryptic digestion, MS	Identification of O-glycoproteins and assessment of their function	Wang et al. (2016)
VVA/Vector labs	40 µl	protein precipice of cell extract, 600 mg	incubation: overnight at 4°C	10 mM HEPES pH 7.5 + metal ions (x3)	boiling (100 °C, 8 min) in Laemmli buffer+ 5% 2BME, 1 mM EDTA, 80 µl	1) SDS-PAGE 2) lectin blotting 3) in-gel tryptic digestion, LC-MS and MS/MS	Characterization of Tn-modified glycoproteins	Hoja-Łukowicz et al. (2018)
**Glycopeptides**								
LCH/GE Healthcare	100 μl	mouse liver tissue, 1 mg HeLa cells, 100 µl (tryptic digest)	tryptic digestion, HILIC, incubation: overnight at 4°C	20 mM Tris- pH 7.45 + metal ions, 400 µl (x4)	1 M acetate, 150 μl, 15min elution (x3)	Endo F3, LC-MS/MS	HILIC and lectin enrichment followed by fragmentation and spectrum refinement method, for the analysis of core fucosylation	Cao et al. (2014)
LCA, PSA, AAL LTL, UEA I/Vector labs AOL-biotin conjugate/TCI America	150 µg/overnight at 4°C	cell culture, 150 µg (tryptic digest)	tryptic digestion incubation: overnight at 4°C	TBS pH 7.4 + metal ions/0.5 ml (x4)	LCA and PSA: 0.2 M α-MM and0.2 M α-MG AAL, LTL, UEA I and AOL: 0.1 M Fuc, 0.5 ml	hSAX LC-MS/MS	analysis of the fucome by combination of enrichment with lectins and hSAX	Zhou et al. (2017)
Con A/GE Healthcare	150 µl	Leafs from Zea mays L. Plant, 0.5 mg	Tryptic digestion, labelling incubation: 1 h	10% ACN, 50 mM ammonium bicarbonate, 300 µl (x5)	0.5 M Man, 75 µl (x2)	PNGase A + PNGase F, LC-MS/MS	N-glycoproteome analysis	Bu et al. (2017)

NOTES: hSAX: hydrophilic strong anion exchange chromatography; N.S: not specified; PBS: phosphate buffered saline; RIPA: radioimmunoprecipitation; SAX: strong anion exchange chromatography; SCX: strong cation exchange chromatography TBS: tris-buffered saline.

As before, the enrichment of glycoproteins or of glycopeptides in dSPE mode were both attempted with commercial lectin-agarose based sorbents. However, compared to the SPE processes mentioned in the previous section, the amount of lectin sorbent used for dSPE appears to be lower. The reported amounts ranged between 60 μl (Song et al., 2014) to 200 μl (Patil et al., 2019) for intact protein enrichment. For glycopeptide enrichment, volumes of about 100 μl are reported. The quantity of treated sample however could greatly vary and range from 600 mg of intact protein (Hoja-Łukowicz et al., 2018) to 150 μg of tryptic digests (Zhou et al., 2017). Lectin dSPE followed by MS analysis has been applied for quantitative and/or qualitative studies of the glycoproteome, with an overall improvement in the number of the identified sites of glycosylation and of the monitoring of the abundance of those glycoproteins that can be used as biomarkers compared to the non-enriched proteome (Song et al., 2014, 2014; Wang et al., 2014, 2016; Bu et al., 2017; Hoja-Łukowicz et al., 2018; Patil et al., 2019). It’s also worthwhile noting some protocols aiming to the further improvement of the coverage of glycosylation capturing. As an example, enrichment at the glycoprotein level, followed by tryptic digestion and re-application of the tryptic peptides to the lectin column and a final hydrazide SPE cartridge was proven more efficient in the total glyco-epitope capture specificity compared to the results obtained when the distinct procedures were performed individually (Li et al., 2015). Other sequential protocols for enrichment included the use of dSPE lectin enrichment with an LCA lectin sorbent and the subsequent application of the sample after desorption to a hydrophilic strong anion exchange chromatography cartridge (hSAX) (Zhou et al., 2017). In this study, 6 fucose specific lectins were evaluated alone or in tandem with the hSAX column in order to assess their ability to enrich fucosylated peptides from different prostate cancer (Pca) cell lines. The sequential enrichment with the LCA lectin sorbent at first followed by hSAX resulted in the higher number of unique glycopeptide and unique fucosylated glycopeptide identifications. Tandem-mass-tags (TMT) labelling and high-resolution accurate-mass LC-MS/MS was further applied for a large-scale analysis of selected Pca cell lines. With this approach, it was seen that the enrichment of glycopeptides without subsequent deglycosylation combined with an MS/MS analysis allowed both the localization of fucosylation sites and the characterization of the structure of the glycans, which is advantageous compared to previous glycan release protocols found in literature. Additionally, the quantitative data in this study led to the identification of novel biomarkers for the differentiation between aggressive and non-aggressive cancer forms. Finally, in another study it was demonstrated that complementary qualitative and quantitative MS data were obtained when biological samples were analysed after either lectin or hydrazide chemistry enrichment performed independently (Song et al., 2014). It should be highlighted though that the MS platforms for data analysis and the MS technology implemented play a decisive role to the extent of the information retrieved (Cao et al., 2014). Overall though, it is evident that there is a tendency to follow more complex protocols with multiple enrichment steps usually in tandem in order to improve the glycoproteome coverage.

## Home-Made Lectin Affinity Sorbents for SPE

Despite the great utility of commercially available sorbents, agarose-based systems have some drawbacks, such as a limited mechanical stability. Therefore, their use in on-line setups, i.e. in coupling with LC-MS or with other affinity columns is quite cumbersome, due to their limited resistance under higher pressures. Agarose also exhibits some limitations concerning biological or chemical stability. Moreover, it is not always easy to find precise information on grafting densities, meaning the amount of lectin incorporated per ml or g of the solid support; therefore, the capacity of commercial sorbents to retain the targeted molecules cannot be easily predicted. While this is not a strong limitation for conducting certain qualitative studies where the analytical performance characteristics of the lectin enrichment process, such as targeted molecule recoveries after the lectin sorbent, is not necessary to be precisely defined, it can be more problematic when a more accurate quantitative study needs to be performed. To overcome these limitations, several academic research laboratories have focused on the development of “home-made” lectin-based sorbents. Table 4 provides a summary of the studies dealing with the development of novel lectin-based affinity sorbents. From this table, it is evident that most of those studies were carried out by using model glycoproteins, mostly in pure media. Additionally, the functionalization of most of the novel sorbents was performed by using Con A, mainly due to the well-known and broad specificity and the relatively low cost of this purified lectin. On the other hand, the choice of the model glycoproteins was based on their known glycosylation profile and thus on their expected affinity towards the used lectins. Some research groups have nevertheless gone as far as the practical application of the developed sorbents, which were hyphenated either off- or on-line with (LC)-MS to analyse biological samples, like serum and plasma, (Madera et al., 2006, 2007; Kullolli et al., 2008, 2010; Selvaraju and El Rassi, 2012; Mann et al., 2013; Selvaraju and Rassi, 2013; Gbormittah et al., 2014b; Liu et al., 2016b; Jonnada and Rassi, 2017; Zheng et al., 2017; Yang et al., 2018). Moreover, the analysis of urine (Feng et al., 2009) or other fluids (Gbormittah et al., 2014a), proteins from cultured cell lines (Sugahara et al., 2015) or human tissue extracts (Peiris et al., 2015) has also been performed. As before, the appropriate processing of the eluted fractions after lectin enrichment, like i.e. buffer exchange and tryptic digestion, was performed to ensure the compatibility with downstream MS-analysis.

**TABLE 4:** Lectin solid phase extraction using home-made sorbents.

lectin(s)	Sorbent	Extraction format, ligand density, grafting yield	Target/volume or amount or concentration	Washing or absorption conditions	Elution conditions	Capacity	Reusability, stability/method performance	Analysis	References
Silica and polymers									
con A	CDAP -activated Sepharose particles	column: x 5 mm–0.75 ml *or* 10 mm–3.0 ml, 2.4–26.4 mg/ml	HRP/1.6 mg pure or 10 ml extract	100 mM acetate pH 6.0 + metal ions	0.1 M α-MM, 20 ml/h	N.S	at least 5 cycles	total aminoacid analysis SDS-PAGE (off-line)	Franco Fraguas et al. (2004)
HPA	NHS activated Sepharose—particles	column: N.S, 2 mg/ml	human tissue/2 mg of protein	N.S	0.25 M GlcNAc	N.S	N.S	IEF, MALDI-MS (off-line)	Peiris et al. (2015)
Con A	1) polymer (Toyopearl), particles: 40–90 μm (pores: 100 nm) 2) Silica, particles: 90–130 μm (pores: 250 nm)	columns:—x 8 mm–vol. 1.5 ml, silica-Con A: 9.8 mg/ml, toyopearl-Con A: 9.4 mg/ml	GOX/1 mg	100 mM acetate pH 5.0 + metal ions	0.1 M α-MM, 0.8 ml/min	Dynamic capacity: Silica Con A: 0.35 mg of GOX/ml Toyopearl Con A: 0.42 mg of GOX/ml	at least for 5 uses	Colorimetric assay	Wen and Niemeyer, (2007)
Con A	1) polymer (Toyopearl)—mPEG-SPA particles	column: 100 × 8mm, 6.5 mg/ml	GOX/250 μl = 1 mg	100 mM acetate pH 6.0 + metal ions	0.1 M α-MM, 0.6 ml/min	Dynamic adsorption capacity: up to 0.4 of GOX ±12/ml	stable against organic solvents and temperature (at least 20 h at 55°C)	UV (on-line)	Wen and Niemeyer, (2011)
RCA	1) polymer (Toyopearl) particles 2) polymer (TSKgel) particles	columns: 1) 50 × 7 mm, 20 mg/g - 69–93% (n = 4) 2) 75 × 7.5 mm, 15 mg/g - 77%	model (glyco)proteins or derivatized glycans/400–500 µg	10 mM phosphate pH 7.2	low pressure: 0.1 M Lac,: 0.8 ml/min HPLAC: (0.005 M Fuc at step gradient,1 ml/min	RCA-TSKgel: 1.67 mg ASF/ml RCA–Toyopearl: 1.7–2.5 mg ASF/ml—(batch exp.)	at least 52 weeks/REC: 86%	UV (on-line)	Cartellieri et al. (2001)
WGA	1) polymer (Toyopearl), particles: 42 µm 2) Silica, particles: 84 µm	columns: 50 × 7 mm 1) 7.8–9.2 mg/g—99.0–99.4% 2) 19.2 mg/g - 99.6%	1) fetuin/500 µl 2) diluted FBS/1 ml (1:10 dilution)	phosphate pH 7.4 ≈ 16 ml	0.2 M GlcNAc ≈4 ml, 0.8 ml/min	WGA–Silica: 5.1 mg of fetuin/ml WGA–Toyopearl: 1.8 mg of fetuin/ml WGA–Toyopearl: 21.6 mg of fetuin in 1 ml of serum	at least 8 months/REC: WGA–Silica: 57% REC: WGA–Toyopearl: 28%	UV (on-line)	Cartellieri et al. (2002)
WGA RCA Con A	1) polymer (Toyopearl) particles,2) Silica particles	Individual columns:50 × 7 mm Con A-silica: 10.7 mg/ml Con A-polymer: 8.4 mg/ml	fetuin and HRP	WGA/Con A: phosphate pH 7.4/acetate pH 6.0 + metal ions	Con A: α-MM, pH 6.0, WGA: GlcNAc, pH 6.0, 0.8 ml/min	purification of 21.6 mg of fetuin/ml of fetal serum	RCA: 71 weeks WGA: 50 weeks Con A: 80 weeks &stability over organic solvent (MeOH 20%)	UV (on-line)	Helmholz et al. (2003)
WGA Con A	1) polymers (Toyopearl, Eupergit C) particles 2) silica particles 3) PEHA-Cel, particles: 84–315 μm, pores: 50–100 nm	Individual columns: 50 × 7 mm–vol: 1 ml, 40 mg/g	GOX/20 mg	WGA: 10 mM phosphate pH 7.2 Con A: acetate pH 6.0 + metal ions	WGA: 0.3 M GlcNAc, Con A: 1 M α- MM, 0.8 ml/min	PEHA-Cel-Con A: 4.8 mg of GOX Toyopearl - Con A: 2.9 mg of GOX	N.S	colorimetric assay	Rosenfeld et al. (2005)
WGA Con A	cellulose-based particles 1) OXY-Cel 2) PEHA-Cel, particles: 200–315 μm	Individual columns:50 × 7 mm, WGA: up to 18 mg/ml, Con A: up to 15 mg/ml	GOX and Fetuin/10 mg	WGA/Con: 100 mM phosphate pH 7.4/100 mM acetate pH 6.0	WGA: 0.3 M GlcNAc Con A: 0.1 M α-MM, 0.5 ml/min	up to 7.4 mg of GOX/ml	REC: up to 93%	UV (on-line)	Aniulyte et al. (2006)
Con A WGA Jacalin	POROS, particles: 20 μm	Multi-lectin column: 30 × 4.6 mm, 15 mg/ml- 95%	1) fetuin and thyroglobulin/2 mg 2) Human plasma/250 μl to 2 mg	25 mM Tris pH 7.4 + metal ions, 5 ml	100 mM acetic acid pH 3.0, 5 ml, 4 ml/min	500 µg of depleted plasma = 100 µl of crude plasma	up to 150 runs/REC: 93%, RSD: 14.7% (total in plasma)	tryptic digestion, LC-MS/MS (off-line)	Kullolli et al. (2008)
Con A WGA Jacalin	POROS, particles: 20 μm	Multi-lectin column: 100 × 4.6 mm	human plasma/50 μl, 1:4 dilution in buffer, depletion (on-line)	25 mM Tris pH 7.4 + metal ions	100 mM acetate pH 4.0,5 x V_c_, 5 ml/min	N.S	at least 200 runs/at least 3 months/REC: ≥95% (plasma fractionation)	desalting column, UV (on-line) or tryptic digestion-LC-MS/MS (off-line)	Kullolli et al. (2010)
Con A WGA Jacalin	POROS, particles: 20 μm	Multi-lectin column: N.S	pancreatic cyst fluid/200 μg, depletion (on-line)	N.S	0.1 M acetic acid pH 2.5	N.S	REC: 84–85%	desalting column (online), SDS gel and in-gel tryptic digestion, LC-MS/MS (off-line)	Gbormittah et al. (2014a)
SNA AAL PHA-L	POROS particles: 20 μm	Multi-lectin column: N.S	renal plasma/20µg, depletion (on-line)	N.S	0.1 M acetic acid pH 2.5	max: 25 ml of plasma	REC: 92% (Depletion and M-LAC)	desalting column (online), LC-MS/(MS) (off-line)	Gbormittah et al. (2014b)
WGA	polymer (TSKgel) particles	column: 50 × 4.6 mm, 14.1 mg/ml—86%	Cell secretome	10 mM Tris pH 7.4	0.01 M GalNAc, 0.3 ml/min	N.S	N.S	HILIC,LC-MS (off-line) or MALDI-MS(MS) (off-line)	Sugahara et al. (2015)
Con A	MALT-Silica particles: 5 µm (pores: 30 nm)	Column 100 × 4.6 mm	model (glyco) proteins derivatized glycans	20 mM Tris pH 7.4 + metal ions, ≈10 ml	0.1 M α-MM ≈ 10 ml, 1 ml/min	N.S	N.S	UV (on-line)	Rathnasekara and El Rassi, (2017)
Con A	silica-Odex particles	Individual column: 150 × 3.0 mm, 16.2 ± 0.06 mg/ml—84%	1) model proteins/50 μg 2) tryptic digest RNase B/10 μl, [C] = 10 mg/ml–100 μg 3) fresh egg and human serum/2 μl, 5 and 10 μl, [C] = 0.074 mg/ml and 0.095 mg/ml	10 mM HEPES pH 7.2 + metal ions, 2 ml	0.1 M α-MM (0–100% in 19 min), 0.2 ml/min	42.7 ± 0.5 mg of OVA/ml	1 year	UV (on-line) or LC-MS/(MS) (off-line)	Liu et al. (2016b)
Con A	Hydrazide activated silica, particles: 7 μm (pores: 30 nm)	column:10 × 2.1 mm, up to 46 mg/g = 10 mg/ml	MUM/5 μl, [C] = 10 μM	(A)500 mM sodium acetate pH 5.0 + metal ions, flow rate: 0.5 ml/min	N.A	N.S	N.S	UV (on-line)	Vargas-Badilla et al. (2019)
Con A AAL	Silica particles: 5 μm (pores: 30 nm)	Individual column:50 × 2.1 mm, Con A: 88 ± 6 mg/g, AAL: 29.4 ± 1.7 mg/g	AGP/20–100 µl, [C] = up to 1.5 mg/ml	Con A (A): 10 mM Tris pH 7.4 + metal ions, flow rate: 0.05 ml/min, temp:50°C, total analysis <20 min AAL (optimal): 10 mM Tris pH 7.4	AAL: 0.001 M Fuc, 0.75 ml/min, -temp: 50°C, total analysis<6 min	Con A: up to 200 µg of AGP–AAL: up to 100 µg of AGP –	Con A: at least 90 times, at least 15 months AAL: at least 80 times, at least 7 months/RSD% (Peak area) < 2%	UV (on-line)	Zhang and Hage, (2019)
Con A AAL	Silica microspheres: 1.6 μm (pores: 100 nm)	Individual columns: 50 × 1 mm, 40 mg/g	1) HRP/up to 75 ug 2) AGP/up to 30 ug 3) human serum/1 μl of diluted (x100) or 40 μg depleted	20 mM phosphate pH 7.4, 600 μl, flow rate: 10 μl/min	Con A: 0.1–0.2 M α-MM, AAL: 0.1 M Fuc- 400 μl, 20 μl/min	silica-Con A for HRP: max 75 μg silica-AAL for AGP: max 20 μg	N.S	tryptic digestion, LC-MS/MS (off-line) or permethylation, glycomic profile: MALDI-MS (off-line)	Mann et al. (2013)
Con A SNA	Silica particles: 10 μm (pores:100 nm)	Individual columns:50 × 1 mm, 8–60 mg/g—>90%	1) RNase B/up to 50 µg 2) mix of model proteins	H_2_0	0.1 M α-MM	RNase B: 50 µg (UV-setup) RNase B: 100 ng (MS-interface)	at least 30 inj. and/at least 6 months/PREC: RSD: 5%	trap column- -LC-MS (on-line)	Madera et al. (2005)
Con A SNA-I UEA-I PHA-L	Silica particles: 10 μm (pores: 100 nm)	Individual columns: 50 × 0.5/1 mm, Con A: 50 mg/g, SNA, UEA-I, PHA-L: 35 mg/g	human serum/5 µl diluted (x5) or 20 µl diluted (x1)	Con A: 10 mM Tris–HCl, pH 7.4 + metal ions SNA, UEA-I, PHA-L, multi lectin: 10 mM phosphate, pH 7.4, 1.5 ml	Con A: 0.1 M α-MM SNA: 0.1 M Lac UEA-I: 0.1 M Fuc PHA-L: 0.4 M GlcNac Multi-lectin: mix of all	N.S	N.S	tryptic digestion, LC-MS/MS (off-line)	Madera et al. (2007)
Con A SNA UEA-I PHA-L	Silica particles: 10 μm (pores: 100 nm)	Individual columns or multi-lectin column: 5 cm × 250 μm, Con A: 50 mg/g, SNA, UEA-I, PHA-L: 35 mg/g	human serum/16 μg, depletion (on-line)	Con A: 10 mM Tris pH 7.4 + metal ions SNA, UEA-I, PHA-L, multi lectin: 10 mM phosphate pH 7.4	Con A: 0.1 M α-MM SNA: 0.1 M Lac UEA-I: 0.1 M Fuc PHA-L: 0.4 M GlcNac Multi-lectin: mix of all	N.S	N.S	trap column, RP fractionation (on-line), in-well- tryptic digestion, LC-MS/MS (off-line)	Madera et al. (2006)
Con A	Magnetic beads - mPGMA: 1.62 μm	MSFB: 1 × 10 cm, 12.5 mg/g	1) IgG/[C] = 0.1–3 mg/ml IgG in human plasma: 25 ml, [C] = 16 mg/ml IgG, 2 h at 25°C	PBS pH 7.4	50% ethyleneglycol, 2 M NaCl, 25 ml, 2 h at 25°C	66.2 mg of IgG/g (standard), 48 mg of IgG/g (in plasma)	N.S	UV (off-line) ELISA (off-line)	Akkaya et al. (2012)
Con A WGA RCA120	Polymer-brush shell hybrid core silica	Individual spin columns, 55.9–80 mg/g	1) model (glyco) proteins/20 µg 2) tryptic glycopeptide enrichment: fetuin (1 μg) mix with BSA (10 µg)–100 µl dilution + 2 h incubation	100 mM Tris pH 7.4 + metal ions, 3 × 400 µl	0.3 M GlcNac, 100 µl x3 (30 min)	N.S	N.S	SDS-PAGE or MALDI-MS (off line)	Pan et al. (2013)
Con A WGA	Polymer PS-MAn-PNIPAm on Nylon sheet, (pores: 737 ± 214 nm)	Individual microscale reactors	1) peptides from AGP/5 μg 2) human plasma/depleted 3 μl (eq. to 1.5 μl of original), tryptic digestion (on-line)	ABC, pH 7.4, DTT + metal ions	ABC pH 8.4, 5 μl/min	N.S	no more than 5 runs/REC (AGP glycopeptides): 83.2 ± 6.0% RSD (peak area): 3.1% (n = 3)	Deglycosylation, LC-MS/MS (on-line)	Yang et al. (2018)
**Monoliths and cryogels**									
Con A	Cryogel: PVA particles—PA beads, particles: 400 ± 50 nm—pores: up to 100 µm	column: x 6.6 mm, up to 25 mg/ml	HRP/8 ml of 100 μg/ml (800 μg)	A: 200 mM phosphate pH 7.5 ≈ 15 ml	1 M gluc ≈5 ml, 1 ml/min	Dynamic capacity: 2.91 ± 0.008 mg/ml	max use: 6cycles	UV (on-line)	Hajizadeh et al. (2012)
Con A	Cryogel: poly (EGDMA-co-MBAAm), (pores: 10–100 μm)	column: N.S	Invertase/1 mg	A: 20 mM acetate pH 5.0, NaCl 1M -2 h	0.1 M ethylene glycol, 20 ml, 2 h, 0.5 ml/min	55.45 mg of invertase/g	up to 10 cycles	UV (off-line) or enzymatic activity	Uygun et al. (2012)
Con A	Cryogel: poly (EGDMA-co-MBAAm), pores: 50–100 μm	column: N.S, 37.97 mg/g	Inulinase/5 mg	A: 100 mM acetate pH 4.0–2 h	0.5 M α-MM, 5 ml, 0.5 ml/min	27.85 mg of inulinase/g	up to 50 cycles	UV (off-line) or enzymatic activity	Altunbaş et al. (2013)
Con A	Cryogel: poly (HEMA-co-EDMA), pores: 2.0 and 0.5 μm	column: N.S, 53.22 mg/g	Amyloglucosidase/4 mg	A: 0.1 M acetate pH 5.0–2 h	0.5 M α-MM, 5 ml, 1 h, 0.5 ml/min	30.50 mg of amyloglucosidase/g	up to 30 cycles/REC: up to 99%	UV (off-line) or enzymatic activity or SDS-PAGE	Bayraktaroğlu et al. (2018)
Con A	Cryogel: poly (HEMA-co-GMA), pores: 10–20 μm	column: N.S	Laccase/3.75 mg	A: buffer pH 3.0, NaCl 1 M, temp 25°C	N.A	7.1 mg of laccase/g	N.S	UV (off-line)or enzymatic activity	Bayraktaroğlu et al. (2020)
Con A	Cryogel: poly (HEMA-co-PEGDA), pores: up to 50 μm	centrifugal unit, N.S	model (glyco)proteins/150 μl, [C] = 0.6–0.8 M—10 min incubation, centrifuge (30 s)	50 mM acetate pH 6.5 + metal ions +10% ACN, 500 µl (x5)	0.2 M α-MM, 150 µl	N.S	at least 5 times	MALDI-MS (off-line)	Krenkova et al. (2015)
Con A	Monolith: cellulose-Cu(II)-IDA, pores: 0.5–2.5 μm	column: 50 × 5.0 mm, 18.9 ± 0.6 mg/ml	1) model glycoproteins: GOX and OVA/50 ml ([C] = 0.2–1.6 mg/ml)	20 mM phosphate buffer + metal ions	up to 0.15 M α-MM—0.2–1.0 ml/min	dynamic capacity: 11.4 ± 1.0 mg of GOX/ml	at least 10 cycles	UV (on/off-line)	Du and Dan, (2018)
Con A RCA-I WGA	Monolith: poly (GMM-co-EDMA)	Individual columns in tandem or alone: 100/50 × 4.6 mm	1) model (glyco)proteins 2) human serum/50 μl of 4x diluted serum	20 mM Tris pH 6.0 + metal ions	WGA:0.1 M GlcNAc, Con A:0.1 M α-MM, RCA: 0.1 M Lac -, 1 ml/min	up to 250 µl of diluted serum	N.S	UV (on-line) or LC-MS/MS (off line)	Selvaraju and El Rassi, (2012)
AAL LTL	Monolith: poly (GMM-co-PETA)	Individual columns in tandem: 50/30 × 4.6 mm	human serum/20 μl of 3x diluted serum, depletion (on-line)	20 mM Tris pH 7.4, 16 ml	0.005 M fuc, 16 ml, 0.8 ml/min	N.S	RSD (peak area) < 12% (n = 2)—LTL/AAL	RPC (on-line), tryptic digestion, LC-MS/MS (off line)	Selvaraju and Rassi, (2013)
LcH	Monolith: functionalized NBE-CL, pores: 4–5 µm	column: 150 × 4.6 mm	model glycoprotein (GOX) with model non glycoprotein (BSA)/50 μl mix	10 mM Bis-Tris pH 6.0 + metal ions, 3 ml	gradient elution (0.2 M α-MM: 0–100%), 10 ml, 1 ml/min	2.2 ± 0.2 mg of GOX/g	N.S	UV (on-line)	Bandari et al. (2013)
WGA Con A	Monolith: poly (GMA-*co*-EDMA)	Individual capillaries alone or in tandem with RPLC: 12/25 cm × 100 μm	model glycoproteins, derivatized glycans	20 mM Bis Tris pH 6 + metal ions ≈ 3–4 CV	Con A: 0.2 M α-MM, WGA: 0.2 M GlcNAc ≈ 3–4 CV, up to 1.67 mm/s flow velocity	N.S	N.S	UV (on-line)	Bedair and El Rassi, (2005)
LCA WGA	Monolith: poly (GMA-co-EDMA)	Individual capillaries alone or in tandem (LC or CEC): 12 cm × 100 μm	model glycoproteins	LAC-LC: 10 mM EDA or 20 mM BisTris, pH 6.0 + metal ions LAC-CEC: wash: 10 mM EDA/DETA/TETA buffer pH 6.0 + metal ions	LCA: 0.2 M α- MM, WGA: 0.2 M GlcNAc, pH 6.0	N.S	N.S	UV (on-line)	Okanda and Rassi, (2006)
Con A	Monolith: poly (GMA-co-EDMA)	capillary: 5 cm × 75 μm, 6.63–11.07 mg/g	Glycopeptides of RNase B/20 μl	ammonium acetate, N.S	ACN:H_2_O 50:50% v/v + 2% AA, N.S	N.S	N.S	MS (on-line)	Bedair and Oleschuk, (2006)
Con A	Monolith: poly (GMA-co-EDMA)/IDA:Cu(II):Con A	capillary: 10 cm × 200 μm	1) glycopeptides of OVA/20 μg 2) human urine/10 μg in diluted in 20 μl	25 mM Tris pH 7.4 + metal ions, 10 µl	ammoniated water pH 10.3–20 μl, 1 μl/min	N.S	at least 10 uses	tryptic digestion, LC-MS/MS (off line)	Feng et al. (2009)
ECL	Monolith - golden nanoparticles: EDMA-DPA-AU-NPs	pipette tips: 20 μl	1) model (glyco)proteins 2) E.coli cell lysate/[C] = 20–80 μg/ml/20 µl	10 mM Tris pH 7.4 + metal ions–20 µl	0.8 M Gal-40 µl, 50 μl/h	N.S	no more than one month	UV (off-line)	Alwael et al. (2011)
LCA Con A RCA	Monolith: poly (NAS-co-EDMA)	Individual columns alone or in tandem: 250 × 1 mm	1) model (glyco)proteins 2) human serum/20 μl of 1:3 dilution	20 Mm Tris pH 6.0 + metal ions, 6 ml	LCA, Con A: 0.1 M α-MM, RCA:0.1M Lac, 2 ml, 0.1 ml/min	N.S	N.S	UV (on-line) or tryptic digestion, LC-MS/MS (off line)	Jonnada and Rassi, (2017)
PNA	Monolith: poly (HEMA-EDMA-PNA-β-CD)	capillary: 3 cm × 530 μm	1) enrichment of IgG galacto-glycopeptides in standard/1 pmol 2) human serum/5 μl of tryptic digest 2) AML cell lysate	85% ACN +0.1% FA, v/v, 0.5 ml	70% ACN +0.1% FA, 10 μl/min	N.S	at least 60 uses, at least 2 weeks	MS (on-line)	Zheng et al. (2017)

Notes: AA: acetic acid; ABG: ammonium bicarbonate; AGP: alpha 1 acid glycoprotein; AML: acute myelogenous leukemia; ASF: asialofetuin; AU-NPs: golden nanoparticle; BSA: bovine serum albumin; ACN: acetonitrile; CD: cyclodexrine; CDAP: 1-cyano-4-(dimethylamino)-pyridinium tetrafluoroborate; CEC: capillary electrochromatography; CL: trimethylolpropane-tris(5-norbornene-2- carboxylate); Cu: copper; DETA: Diethylenetriamine; DPA: 2,2-dimethoxy-2-phenylacetophenone; DTT: dithiothreitol; EDA: ethylenediamine; EDMA: ethylene glycol dimethacrylate; FA: formic acid; FBS: fetal bovine serum; GMA: glycidyl methacrylate; GMM: glycerylmethacrylate; GOX: glucose oxidase; HEMA: hydroxyethylmethacrylate; HRP: horseradish peroxidise; HSA: human serum albumin; IDA: iminodiacetic acid; IEF: isoelectric focusing; IgG: immunoglobulin G; LA: lipoic acid; LAC: lectin affinity chromatography; LC: liquid chromatography; LR: linear range; MALDI: matrix-assisted laser desorption/ionization; MALT: maltose; MBAAm: N,N′-methylene-bisacrylamide; MSFB: magnetically stabilized fluidized bed; mPEG-SPA: monomethoxy poly(ethylene glycol) succinimidyl propionate; MS: mass spectrometry; MUM: 4-methylumbellipheryl α-D-mannopyranoside; NAS: N-acryloxysuccinimide; NBE: norborn-2-ene; NHS: N-hydroxy-succinimide; ODex: oxidized dextran; OVA: ovalbumin; OXY-Cel: periodate activation cellulose; PA: porous adsorbent polymer beads; PEGDA: polyethylene glycol diacrylate; PEHA-cel: pentaethylenehexamine-cellulose; PHEMA: poly(2-hydroxyethyl methacrylate); PETA: pentaerythritol triacrylate; PREC: precision; PSHSM: polymer-brush shell hybrid silica; PS-MAn-PNIPAm: polystyrene-maleic anhydride-nisopropylacrylamide; PVA: polyvinylalcohol; REC: recovery; REP: repeatability; RNase B: ribonuclease B; RPC: reverse phased column; SDS: sodium dodecyl sulphate; TETA: triethylenetetramine.

### Silica- and Polymer-Based Particles

Silica-based materials have been traditionally used in chromatography, as they can withstand higher pressure and are available in various particle and pore sizes. Additionally, the surface chemistry of silica can be easily altered to facilitate the grafting of biomolecules (Schiel et al., 2006). Similarly, polymeric supports are rigid materials, which are available in various chemistries, pore and particle sizes, are stable in a wider pH range than silica and can potentially exhibit reduced non-specific interactions. In their application in lectin affinity studies, supports with large particle diameter of up to 315 μm (Rosenfeld et al., 2005) have been used, which facilitates the percolation under gravity or their incorporation in low-pressure set-ups. On the other hand, smaller particles with diameter as low as 1.6 μm (Mann et al., 2013), which necessitate the use of high-pressure pumping systems, have been reported. The majority of the studies describes the use of commercial polymer or silica particles that have been functionalized with lectins using widely described and well characterized procedures for the grafting of biomolecules.

Moreover, certain functionalization processes have been followed, like aldehyde modification (Madera et al., 2005, 2006, 2007), tresylchloride (Helmholz et al., 2003) or hydrazide activation (Vargas-Badilla et al., 2019) or incorporation of spacer molecules (Rosenfeld et al., 2005) before lectin immobilization. In addition to the use of commercial particles, other supports have been developed, like hydrid silica-polymer particles (Pan et al., 2013), poly (ethylene glycol)-(PEG)ylated-polymeric particles (Wen and Niemeyer, 2011), oxidized-dextran-silica (Liu et al., 2016b), maltose-silica (Rathnasekara and El Rassi, 2017) and modified cellulose materials (Rosenfeld et al., 2005; Aniulyte et al., 2006). These modifications aimed to improve the lectin immobilization process, increase the stability of the modified support, diminish non-specific interactions and improve the capacity of the sorbent towards the analyte. For example, the incorporation of a 1,4-butane-diol-diglycidyl ether spacer molecule in silica particles and of pentaethylenehexamine in cellulose (PEHA-cel) leaded to better immobilization kinetics of the lectins on these supports as opposed to two other commercial non-modified supports. Additionally, the dynamic capacity of the lectin-functionalized PEHA-cel support towards glucose oxidase (GOX) was 4.8 mg of GOX per ml of sorbent, as opposed to unmodified particles, which exhibited a corresponding value of 2.9 mg/ml (Rosenfeld et al., 2005). Additionally, the comparison of the reported lectin densities of the modified supports in this aforementioned study, which are up to 40 mg/g of support, are quite high compared to previous studies, where no modifications were performed and densities were below 20 mg/g (Cartellieri et al., 2001, 2002). Finally, it should be noted that the use of small silica particles increased grafting densities up to 88 mg of lectin/g, due to a higher surface area available for immobilization (Zhang and Hage, 2019). A lectin density of up to 80 mg/g was also achieved using a polymer-brush shell silica core (PSHSM) support for lectin immobilization. This was attributed to the extended surface available for immobilization because of the brushes of the polymer protruding out of the core of the silica (Pan et al., 2013). In this later study, the lectin-agarose counterpart was proven less effective in the enrichment of target glycopeptides both from a quantitative and qualitative aspect.

These home-made lectin sorbents were mostly packed in columns with an i.d. of 4.6 mm (Kullolli et al., 2008, 2010; Sugahara et al., 2015; Rathnasekara and El Rassi, 2017) and up to 8 mm (Wen and Niemeyer, 2007, 2011). Flow rates of typically 0.8–1 ml/min were implemented by using a low- or high-pressure pumping system. For example, Kullolli et al. developed a high-pressure lectin affinity purification device with grafted particles of POROS polymer of 20 µm diameter packed in a 30 × 4.6 mm column and combined off-line with LC-MS/MS analysis (Kullolli et al., 2008). In this study, POROS was functionalized with 3 different lectins with good grafting densities (15 mg of lectin per ml of sorbent) and the produced lectin sorbents were mixed together to be packed in a multi-lectin column. This column showed a great capacity towards depleted plasma, as up to 500 μg of sample did not saturate the column. The elution was performed with acidic conditions using a solution of 100 mM acetic acid pH 3.8 of low viscosity instead of a saccharide solution thus allowing the use of a high flow rate of 4 ml/min that led to a fast analysis. Results showed that compared to the conventional lectin agarose column, the multi-lectin POROS column offered an increased binding capacity towards the glycoproteins of plasma with a much lower total analysis time. The same format was used in a subsequent study, coupling the lectin column in series between an upstream depletion column and a downstream desalting column in a fully automated setup prior to off-line LC-MS analysis (Kullolli et al., 2010). Finally, the same platform was used to analyse the proteome of pancreatic cyst fluid (Gbormittah et al., 2014a) and renal plasma (Gbormittah et al., 2014b) for biomarker identification in related malignancies.

Apart from the aforementioned large-scale formats, smaller scale systems have also been developed. Madera et al. conducted a series of studies with 0.25–1 mm i.d. lectin microcolumns with operational flow rates between 5 and 50 μl/min. In the first study (Madera et al., 2005), aldehyde-modified silica particles were coupled with different lectins with high coupling yields of more than 90% and lectin densities of up to 60 mg of lectin per g of support. As seen in Table 4, this value is quite high in the overall range of 9.2–88 mg/g reported. The direct hyphenation of a lectin microcolumn with a desalting trap column and the direction of the eluate towards the nanoLC-MS setup allowed the efficient enrichment and analysis of glycopeptides from trypsin-digested model glycoproteins. Additionally, it should be noted that elution was performed with the competitive saccharide, since the presence of the desalting trap column allowed its removal before LC-MS analysis. After this development achieved using pure standard of glycoproteins, this set-up was slightly modified and was applied to the analysis of more complex samples. Indeed, 16 μl of human serum were depleted and subsequently enriched separately with individual lectin microcolumns directly coupled with the desalting column. Then, an RP fractionation column connected after the desalting one was used to collect fractions, which were then treated with trypsin to be analysed by off-line LC-MS/MS (Madera et al., 2006). Diluted human serum was analysed with a similar format either with single or multi-lectin microcolumns and analysed after tryptic digestion in an off-line step. In this experiment, it was seen that the combined results of individual enrichment with each lectin microcolumn offered overall a greater coverage of the glycoproteome as compared to mixing all the lectin sorbents together in the multi-lectin format, while minimal amount of sample was needed, indicating an increased sensitivity of the miniaturized setup (Madera et al., 2007).

### Monolithic Affinity Sorbents

As illustrated in Table 4, the immobilization of lectins on monoliths has also been performed (Bedair and El Rassi, 2005; Bedair and Oleschuk, 2006; Okanda and Rassi, 2006; Feng et al., 2009; Alwael et al., 2011; Selvaraju and El Rassi, 2012; Bandari et al., 2013; Selvaraju and Rassi, 2013; Jonnada and Rassi, 2017; Zheng et al., 2017; Du and Dan, 2018). A monolith is a one-piece continuous porous sorbent containing macro- and mesopores. Those sorbents can be advantageous since they can exhibit a high porosity and thus a high permeability generating lower back pressures (Dziomba et al., 2017; Li et al., 2017). According to their chemistry, they can be classified into organic and inorganic. Organic monolithic supports are synthesized by the *in situ* polymerization of a homogenous mixture of individual monomers, which can also act as cross-linkers, usually in the presence of an initiator and a mixture of porogenic solvents. In addition, cryogel monoliths have been functionalized with lectins (Hajizadeh et al., 2012; Uygun et al., 2012; Altunbaş et al., 2013; Krenkova et al., 2015; Bayraktaroğlu et al., 2018, 2020). Similarly, cryogel monolith synthesis necessitates the polymerization of individual monomers while the reaction is performed at low temperatures in semi-frozen conditions (Lozinsky, 2008). In most of the cases, methacrylate-based monomers were used, mainly with the selection of ethylene glycol dimethacrylate (EDMA) as cross-linker (Bedair and El Rassi, 2005; Bedair and Oleschuk, 2006; Okanda and Rassi, 2006; Feng et al., 2009; Alwael et al., 2011; Selvaraju and El Rassi, 2012; Selvaraju and Rassi, 2013; Jonnada and Rassi, 2017; Zheng et al., 2017). Other reports include the preparation of a lectin-functionalized cellulose-based monolith (Du and Dan, 2018), of a norborn-2-ene-trimethylolpropane-tris(5-norbornene-2-carboxylate) (NBE-CL) monolith (Bandari et al., 2013) and of a composite combining polyvinylalcohol (PVA) particles in a cryogel after a cryogelation process (Hajizadeh et al., 2012). The resulting materials were highly porous, with macroporous channels up to 100 μm thus ensuring the preparation of a sorbent with a high permeability.

In large scale systems, monolithic solid supports and cryogels are available as columns with i.d. between 4.6 mm (Selvaraju and El Rassi, 2012; Bandari et al., 2013; Selvaraju and Rassi, 2013; Jonnada and Rassi, 2017) and 6.6 mm (Hajizadeh et al., 2012). In an interesting study, three different lectins were immobilized in three different polyglycerylmethacrylate (GMM)-co-EDMA monolithic columns, each one with an i. d. of 4.6 mm, and were used on-line and in tandem followed by an off-line LC-MS analysis of human serum. Results indicated that the order in which the individual columns are placed can affect the coverage of the glycoproteome (Selvaraju and El Rassi, 2012). In a later study, this setup was extended, for the creation of an automated platform by coupling on-line four different depletion columns followed by two different poly (GMM-co-PETA) monolithic columns functionalized with either the *Lotus tetragonolobus* lectin (LTL) or the AAL lectin and a final RP fractionation column, all placed in tandem and all having an 4.6 mm i.d (Selvaraju and Rassi, 2013). Briefly, this fully automated multicolumn system was controlled by HPLC pumps and switching valves in order to immediately direct the eluted fraction from one column to the next one without any off-line sample handling. The final protein fractions from the RP column of the on-line format were collected in specific time intervals before additional off-line vacuum drying, tryptic digestion and LC-MS/MS. This approach allowed the enrichment of fucosylated proteins from human sera with minimal sample losses and good reproducibility of the overall procedure. In a later study, a micro format with N-acryloxysuccinimide (NAS)-co-EDMA (NAS-co-EDMA) individual monoliths *in situ* polymerized in a 1 mm i.d. column and functionalized with 3 different lectins independently was implemented. The individual columns were again used in tandem and it was once more noticed that the order in which they were placed affected the final enrichment (Jonnada and Rassi, 2017).

Additionally, as seen in Table 4, in most cases cryogels have been incorporated in columns. Only one study reports the use of a small scale format with a poly (2-hydroxyethylmethacrylate)-co-polyethyleneglycol diacrylate (HEMA-co-PEGDA) cryogel placed in a spin column and functionalized with Con A (Krenkova et al., 2015). This allowed the use of a smaller volume of the sample and elution buffer (150 μl). Lectin-functionalized cryogels were mainly used for absorption studies for the isolation of some enzymes for further industrial applications, like laccase or amyloglucosidase. As mentioned before, cryogels are macroporous structures and therefore they can exhibit lower pressure drop and minimized diffusion resistance; as a result, they can be advantageous for scaling-up the “clean-up” processes of these enzymes compared to classical chromatographic processes.

Moreover, one common practice followed is the *in situ* polymerization of the monolithic supports in fused-silica capillaries, with a narrow i.d. in the μm scale range (75–530 μm), followed by their functionalization with lectins. As expected, the small internal diameter of the capillary allows the fabrication of miniaturized systems that is accompanied by low flow rates in the μl/min scale. Additionally, the consumption of buffers and sample is minimized as it is limited to a few μl. Characteristically, the enrichment of 1.08 pmol (43 ng) of a model glycoprotein could be achieved with an extended injection time of 30 min of a highly diluted sample (5 × 10^−8^ M) through the lectin-based monolith (Bedair and El Rassi, 2005). This proved the good affinity of the lectin-based monolith for the targeted protein, as a volume corresponding to 15.8 Vc, *i.e.* 21.7 μl was percolated through the sorbent. In another format with a poly (HEMA-EDMA)-PNA-β-cyclodextrin (CD) monolith coupled directly to MS, the detection of 5 fmol of the IgG tryptic digest spiked in serum was achieved (Zheng et al., 2017).

Other miniaturized systems were also proven quite efficient for the enrichment of low quantities of biological samples this time. For example, 10 µg of urinary proteins diluted in 20 µl were enriched with a Con A-based monolithic capillary of 10 cm × 200 μm i.d. (Feng et al., 2009). The results after MS analysis showed a better reproducibility and a two times increment in the identified glycoproteins as compared to the agarose-based lectin sorbent. Another interesting miniaturized format included the incorporation of only 20 µl of a dimethacrylate monolith with attached golden nanoparticles and functionalized with an *Erythrina cristagalli* (ECL) lectin in pipette tips (Alwael et al., 2011). This also favoured small sample consumption, as only 20 μl of the sample were used and eluted with 40 μl of buffer. In general, these practices can be proven beneficial when low quantities of biological samples are available.

### General Advantages of “Home-Made” Lectin-Based Sorbents for SPE

The diversity of homemade sorbents is evident from the variety of phase chemistry studied and the resulting wide range of grafting densities. Commercially available lectins immobilized on agarose usually have grafting densities of 2.5–8 mg of lectin per ml of sorbent. As seen in Table 4, when this value is expressed in mg of lectin per ml of sorbent, the range of densities for home-made sorbents was 6.5–26.4 mg/ml, with only a low value of 2 mg/ml given for a Sepharose-lectin functionalized sorbent (Franco Fraguas et al., 2004). It should be mentioned though that, for home-made sorbents, the grafting density is given usually in mg of lectin per g of sorbent and in this respect the range of densities was 9.2–88 mg/g. In this case it is difficult to make a direct comparison with the grafting densities of the commercial lectin sorbents, but it is indicative that they can be tailored and optimized.

As mentioned before, the characterization of the sorbents was mostly done with model proteins. The range of the capacity towards them was from 0.35 to 42.7 mg/ml. Better capacities were achieved when a functionalization process of the support was included before the lectin immobilization. For example, the highest value of 42.7 mg/ml was achieved with a modified silica with oxidized dextran as a spacer (Liu et al., 2016b). Concerning cryogels, however, the capacities towards enzymes were mentioned in mg/g and the range was between 7.1 and 55.4 mg/g. Therefore, even though a direct comparison cannot be made with the other sorbents, a good potential in capturing the targeted glycan moieties can be seen. Additionally, a direct comparison in terms of capacity with commercial sorbents cannot be made, as in those studies mainly biological samples were tested. However, in a few studies, a comparison of the home-made sorbent with the commercial agarose-based one was performed. (Helmholz et al., 2003; Aniulyte et al., 2006; Ferreira et al., 2011; Pan et al., 2013). Characteristically, the capacity of a PEHA-CEL-WGA support towards GOX was 7.4 mg/ml, whereas the corresponding value for the WGA-agarose sorbent was 4.4 mg/ml. Therefore, an improvement in capacity was achieved with the home-made support compared to commercial lectin-agarose.

**FIGURE 3:**
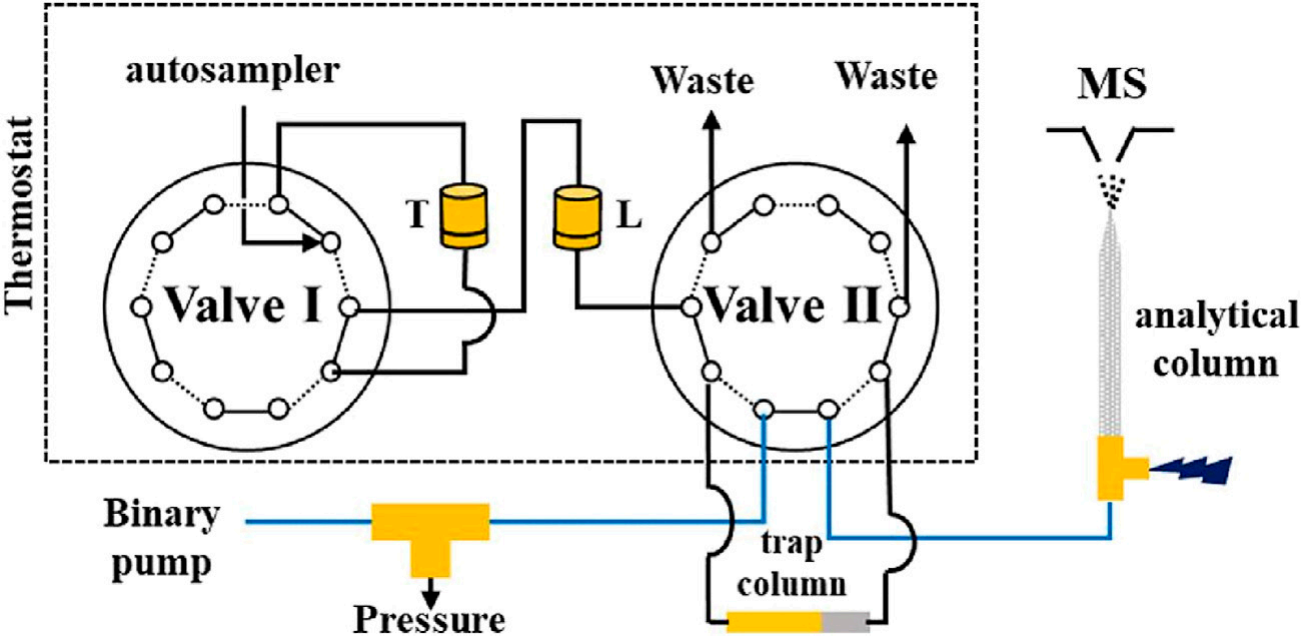
Configuration of the serial TPPMRs for online proteolysis and glycopeptide enrichment prior to nLC-ESI-MS/MS, Setup configuration: *Operational position 1*: on-line digestion and glycopeptides enrichment, Valve positions: I: solid and II: dotted lines. *Operational position 2:* PNGase F injection and loading to trap column, Valve positions: I: dotted and II: solid lines. *Operational position 3:* LC/MS run of deglycosylated peptide and re-conditioning of TPPMR, Valve positions: I: solid and II: dotted lines. TPPMR: thermoresponsive porous polymer membrane reactor. nLC-ESI-MS/MS: nanoflow liquid chromatography-tandem mass spectrometry. T: Trypsin immobilized TPPMR. L: Lectin immobilized TPPMR. Reprinted with permission from Yang et al., Anal. Chem. 2018, 90, 3,124–3,131, Copyright ^©^ 2018, American Chemical Society.

As previously mentioned, another important characteristic of those home-made sorbents is their pressure resistance allowing the possibility for their on-line coupling in high-pressure workflows with LC-MS and the development of miniaturized systems. Among the most interesting formats, one can notice the direct hyphenation of a monolithic-lectin capillary to an MS source, serving both as enrichment sorbent and emitter (Zheng et al., 2017). Since the eluate was directed directly to the MS source, elution buffers were organic to ensure compatibility. Results of this study showed that the enrichment with the lectin monolith led to higher detectability and to an increase of the relative abundance of the targeted glycopeptides compared to the data obtained without this process. Finally, the direct and automated coupling of a lectin sorbent to LC-MS including two enzymatic digestion steps has been recently described (Yang et al., 2018). This device comprises a trypsin thermoresponsive porous polymer membrane reactor (TPPMR) coupled upstream with an immobilized lectin sorbent for the trapping of glycopeptides, which are further directed to the nanoLC system with an elution solution containing an additional deglycosylation enzyme (Figure 3). With this setup, the analysis of 1.5 μl of plasma was automatically performed, indicating that minimal volume of biological samples can be analysed with a sufficient sensitivity. Results showed that when using the automated setup a higher number of glycopeptides/glycoproteins was identified as compared to the off-line in solution tryptic digestion, lectin enrichment and degylcosylation followed by LC-MS analysis.

An additional advantage is that those lectin-based sorbents coupled with further downstream procedures either off- or on-line are automated and faster compared to conventional approaches, as the ones described in the majority of the studies in Table 2. In those later studies, increased sample handling and high elution volumes were required, which are known to impact negatively the quantitative and qualitative data obtained, i.e. by sample losses, induced protein oxidation or proteolysis, loss in sensitivity due to dilution etc. Additionally, the increased analysis time of conventional techniques renders the throughput analysis of multiple samples more problematic and time consuming. In this regard, the developed platforms described above offer a very attractive solution and very promising results in mining the glycoproteome.

It should be noted that most of the home-made sorbents functionalized with lectins exhibited increased stability and were reusable for multiple times. In Table 4, a range of 5–90 uses is reported without a loss in the enrichment ability of the sorbents. Indeed the poly (HEMA-EDMA-PNA-β-CD) monolith was used over 60 times over a time frame of 2 weeks and did not lose its efficiency in glycopeptide capturing, even though elution was performed with an organic-based solution (Zheng et al., 2017). Additionally, in most of the cases a long-term storage was achieved, ranging from a few weeks to even more than a year. In two other studies (Helmholz et al., 2003; Wen and Niemeyer, 2011), it was also reported that a particle polymeric (Toyopearl)-Con A sorbent and a PEGylated polymeric-Con A sorbent were also both stable with methanol. The reusability of a POROS-lectin column for up to 150 runs was also reported when elution was performed in acidic conditions (Kullolli et al., 2008). Finally, a copper Cu(II)-iminodiacetic acid (IDA)-cellulose monolith that can be regenerated was designed (Feng et al., 2009), by forming a reversible complex with Con A through chelation. In this support, the chelated lectin could be easily removed and replaced by a new active one if a loss in efficiency is observed.

**FIGURE 4:**
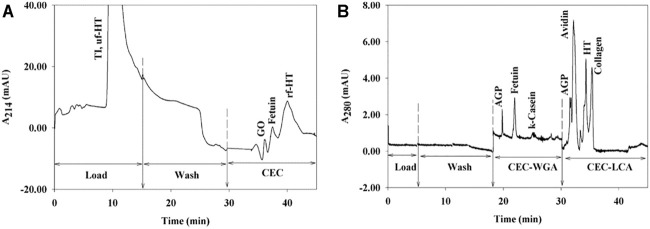
Electrochromatogram of **(A)** GO, fetuin, and HT in the presence of nonglycosylated TI, obtained on WGA immobilized on a monolithic column by a three-step process and of **(B)** AGP, HT, collagen, k-casein, avidin, and fetuin, obtained on coupled monolithic lectin columns (order WGA → LCA). HT: human transferring. TI: soybean trypsin inhibitor. AGP: a1-acid glycoprotein. Reprinted from Okanda, F.M. and El Rassi, Z., 2006, ELECTROPHORESIS, 27: 1,020–1,030 with permission from John Wiley and Sons.

It should be highlighted that in all conducted studies, a good specificity of the sorbent towards the expected glycosylation pattern is mentioned. This was mostly supported by the general observation of the qualitative recovery after elution. The preparation of control sorbents, like for example the corresponding ones without the grafting of lectin (Rosenfeld et al., 2005; Wen and Niemeyer, 2011; Uygun et al., 2012; Altunbaş et al., 2013; Bandari et al., 2013; Pan et al., 2013; Zheng et al., 2017; Demir et al., 2018; Du and Dan, 2018; Bayraktaroğlu et al., 2020) was an additional confirmation of the good specificity of the functionalized material. In all those studies, a good separation between the non-retained and retained forms due to their affinity with the lectin was demonstrated. In one study (Okanda and Rassi, 2006), this partition between non-glycosylated and glycosylated proteins was on-line monitored and glycosylated proteins were further on line separated in capillary electrochromatography (CEC) as shown in Figure 4. More specifically, WGA and LCA lectins were grafted on poly (GMA-co-EDMA) monoliths that were synthesized *in situ* in 12.5 cm × 100 μm capillaries. Those two lectin sorbents were coupled alone (Figure 4A) or in series (Figure 4B) to CEC. In the obtained electrochromatograms, not only the glycoproteins are present in the elution fraction but also a clear separation between them was observed.

### Home-Made Lectin-Based Sorbents for Dispersive Solid-Phase Extraction

Home-made lectin-based sorbents based on polymeric particles (Helmholz et al., 2003; Yavuz et al., 2004; Rosenfeld et al., 2005; Aniulyte et al., 2006; Wen and Niemeyer, 2011, 2011; Hajizadeh et al., 2012; Demir et al., 2018) were applied also in dSPE, as summarized in Table 5. Additionally, dSPE processes can also be performed with lectin grafted to magnetic particles (Ferreira et al., 2011; Akkaya et al., 2012; Qin et al., 2014; Idil et al., 2015; Miura et al., 2018; Waniwan et al., 2018). This approach can simplify the separation process between the bound and the unbound fraction, as it necessitates the use of a magnet to separate both fractions rather than by a tedious centrifugation step. As mentioned also in Dispersive Solid Phase Extraction With Commercial Lectin-Based Sorbents, elution volumes are in general lower. As it can be seen in Table 5, high grafting densities of lectins on magnetic nanoparticles have been obtained. Indeed, grafting densities between 80–120 mg (Ferreira et al., 2011) and 290–315 mg (Waniwan et al., 2018) of lectin per g of support were reported. Compared to the other values found in Table 5, which are not more than 40 mg/g, these grafting densities are quite high. This can be explained by the use of nanoparticles, which provide high surface areas for immobilization. Additionally, a comparison of these nanoparticles-based sorbents with the commercial agarose-beads showed a 5 times increment of the affinity towards model glycoproteins (Ferreira et al., 2011). However, when analysing biological samples, adsorption of non-glycosylated forms was observed in both studies, which may correspond to non-specific interactions of these compounds with the nanoparticle surface.

**TABLE 5:** Lectin dispersive solid phase extraction using home-made sorbents.

lectin(s)	Sorbent (particle or pore size)—amount or dimensions	Ligand density/grafting yield	Target/amount	Incubation (I) and washing (W) conditions	Desorption conditions	Capacity	Analysis	References
Con A WGA MAL	MNP-Fe_3_O_4_(diameter: 14 nm)—1 mg	80–120 mg/g	1) model proteins 2) human serum, saliva, and urine/50 μg, [C] = 250 μg/ml in 200 µl of binding buffer	I: 5 min at 4°C (Con A) or 25°C (WGA, MAL),W: 20 mM Tris pH 7.4 + metal ions, 3 × 200 μl	0.5 M α-MM (Con A) (3 × 200 μl)	46 ± 4 mg of fetuin/g	tryptic digestion, LC-MS/MS (off-line)	Ferreira et al. (2011)
Con A	Magnetic beads—mPGMA-HDMA—(diameter: 3.5 μm)–10 mg	37.3 mg/g	human serum (total PSA and free PSA)/1 ml, diluted x10 or x20	I: 2 h	0.1 M Man, 1 ml, 2 h at 25°C	91.2 ng of PSA/g	SDS-PAGE ELISA	Idil et al. (2015)
Con A	Magnetic particles	N.S	human cells/2 mg of total proteins - diluted at 600 µl	I: 3 h W: 0.1 M Tris pH 7.4 + metal ions + tween x3	0.1 M α-MM with protease inhibitor, 300 μL, 1 h at RT	N.S	tryptic digestion, LC-MS/MS (off-line)	Qin et al. (2014)
ECA (biotinylated)	DynaBeads^®^ M-280 Streptavidin	N.S	human plasma/100 µl (depleted from albumin)	I: overnight at 4°C W: PBS-T x1 + 0.2 M GlcNAc x1 at 4°C for 30 min	0.2 M Lac,1 h at 4°C	N.S	tryptic digestion, (Glu-C),LC-MS/(MS) (off-line)	Miura et al. (2018)
Con A ALL SNA	MNP-Fe_3_O_4_ (diameter: 10 nm)-200 μg	290–365 mg/g	human cells/150 μg of tryptic peptides diluted in 300 μl	I: vortex 1 h W: PBS x1 + PBS/30% ACN x2	50:50 H_2_O:ACN +0.1% TFA	N.S	(deglycosylation), LC-MS/MS (off-line)	Waniwan et al. (2018)
Con A	Cryogel (PHEMA)	up to 10 mg/g	Invertase	I: 0.1 M acetate (optimal pH 5.0), 2 h at 25°C	N.A	107 mg/g (theoretical)	UV (off-line) enzymatic activity	Yavuz et al. (2004)
Con A	hydrogel membrane of p (HEMA-EDMA)- diameter: 0.6 cm	3.52 mg/g	IgG/1 ml [C] = 1.5 mg/ml	I: pH < 5, temp. up to 37°C, 30 min, low ionic strength	N.A	26.81 mg/g	UV (off-line)	Demir et al. (2018)
Con A	polymer particles (Toyopearl) (diameter: 40–90 μm) Silica particles (diameter: 90–130 μm)—0.2 ml or 1 ml	silica-Con A: 11.7 mg/ml Toyopearl-Con A: 11.8 mg/ml	GOX/4 ml or 1 ml, [C] = 0.1–2 mg/ml	I:sample (4 ml) with 0.2 ml of sorbent, 15 h, in 0.1 M acetate pH 5	0.1 M α-MM, 20 ml	total absorption capacity: Silica Con A: 2.18 ± 0.14 mg of GOX/ml Toyopearl Con A: 4.88 ± 0.04 mg of GOX/ml	UV(off-line)	Wen and Niemeyer, (2007)
Con A	PVA particles (diameter: 400 ± 50 nm) in 1 ml cryogel (8 × 13 mm)	up to 25 mg/ml	HRP/5 ml, [C] = 100 g/ml	I: 0.1 M phosphate pH 7.5, overnight at 6–7°C	N.A	1.25 ± 0.05 mg of HRP/ml	UV (off-line)	Hajizadeh et al. (2012)
Con A	polymer particles (Toyopearl) - mPEG-SPA, 0.1 g–0.15 ml	up to 9.9 mg/ml	GOX/5 ml	I: 0.1 M acetate buffer with metal ions, 15 h at 25°C	N.A	11.4 mg of GOX/ml	UV (off-line)	Wen and Niemeyer, (2011)
WGA RCA Con A	polymer particles (Toyopearl)–305 mg Silica particles - 405 mg	Con A-silica: 10.7 mg/ml Con A-polymer: 8.4 mg/ml	GOX/20 ml, [C] = 0.50–0.53 mg/ml	I: phosphate buffer pH 6.5, 20 h at 25°C	N.A	Theoretical Con A-silica: 59 mg of GOX/ml Con A-polymer: 47 mg of GOX/ml	colorimetric assay	Helmholz et al. (2003)
WGA Con A	1) polymer particles (Toyopearl, Eupergit C) 2) silica particles 3) PEHA-Cel particles (diameter:84–315 μm,pores: 50–100 nm)	40 mg/g	GOX, fetuin	I: WGA: 0.01 M phosphate pH 7.2 I: Con A: acetate pH 6	N.A	theoretical/experimental PEHA-Cel (Con A): 23.5/9.7 mg of GOX/ml Toyopearl (Con A): 13.9/8.1 mg of GOX/ml	colorimetric assay SDS-PAGE	Rosenfeld et al. (2005)
WGA Con A	cellulose particles 1) OXY-Cel 2) PEHA-Cel, (diameter: 200–315 μm), 300 µg	PEHA (1.1)-Cel (Con A): 15 mg/ml OXY-Cel (Con A): 9 mg/ml	GOX, 4 ml [C] = 0–6 mg/ml	I: 0.1 M acetate pH 6, NaCl 0.5 M, 3 h at 25°C	N.A	theoretical/experimental OXY-Cel (Con A): 14.1/11.3 mg of GOX/ml PEHA (1.1)-Cel (Con A): 23.5/14.0 mg/ml	colorimetric assay	Aniulyte et al. (2006)

Notes: HDMA: 1,6-diaminohexane; IgG: immunogloboulin; LA: lipoic acid; MNP: magnetic nanoprobes; mPGMA: poly(glycidyl methacrylate); PHEMA: poly(2-hydroxyethyl methacrylate); PSA; prostate specific antigen.

## Conclusion and Perspectives

The development of novel lectin-based affinity sorbents is of major interest, especially in proteomic research, since their use can greatly contribute to the characterisation of glycosylation. This is an important area of study to date, as illustrated by the significant demand in the area of characterisation of therapeutic antibodies. The availability of a large number of commercialized lectin affinity sorbents certainly gave the opportunity to several research groups to readily use them in order to characterize biological samples. Indeed, a wide range of studies with commercialized-lectin affinity sorbents has been conducted, with extensive results in terms of the thorough study of the glycoproteome.

Despite the great applicability and the ease in the use of commercialized sorbents, home-made functionalized affinity supports can be advantageous in many aspects. For example, an improvement in grafting density of the lectin to the support and/or of the capacity towards targeted glycoforms can be achieved. Moreover, an easier adaptation in automated on-line and/or miniaturized analytical set-ups can be done. Those supports can even be proven more cost-effective especially in the miniaturized systems, in terms of the usage of consumables and sample amounts. These reasons were the impetus for many research groups to develop and characterize their own lectin functionalized sorbents. However, the applicability of those supports in real biological samples still has to be more thoroughly explored. The standardization of the procedures for immobilization and a more diligent proof of the better performance of the home-made supports are important actions towards this direction.

It is important to point out that parameters like for example the accessibility of the lectin, the capacity of the sorbent, the sample breakthrough volumes and the reusability of the supports are highly important in order to develop robust and quantitative methods. In the case of home-made sorbents, great efforts have already been made to define of those characteristics. However, there is certainly a lot of space for a better understanding of those parameters.
